# Microanatomic Distribution of Myeloid Heme Oxygenase-1 Protects against Free Radical-Mediated Immunopathology in Human Tuberculosis

**DOI:** 10.1016/j.celrep.2019.08.081

**Published:** 2019-09-17

**Authors:** Krishna C. Chinta, Md. Aejazur Rahman, Vikram Saini, Joel N. Glasgow, Vineel P. Reddy, Jeremie M. Lever, Shepherd Nhamoyebonde, Alasdair Leslie, Ryan M. Wells, Amie Traylor, Rajhmun Madansein, Gene P. Siegal, Veena B. Antony, Jessy Deshane, Gordon Wells, Kievershen Nargan, James F. George, Pratistadevi K. Ramdial, Anupam Agarwal, Adrie J.C. Steyn

(Cell Reports *25*, 1938–1952.e1–e5; November 13, 2018)

In the original version of this article, an error in Figure 4E was noted after publication. A version of the upper left panel of Figure 4D was inadvertently used as the upper left panel of Figure 4E and incorrectly identified as Trichrome staining. This error occurred during the preparation of figures and went unnoticed during the review process and during preparation of final publication.

The original and revised Figure 4E is shown below, with the correct Trichrome stain image in the upper left panel. This correction does not alter any of the findings or conclusions of the study.

The authors regret this error.

**Figure 4E:**
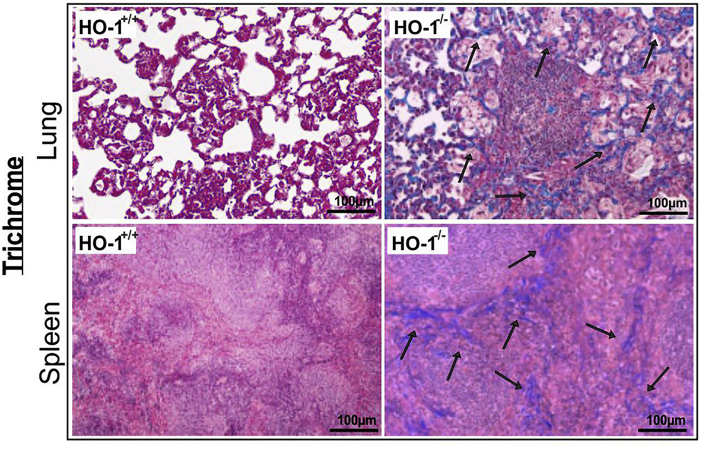
HO-1^−/−^ Mice Are More Susceptible to *Mtb* Infection (corrected)

**Figure 4E:**
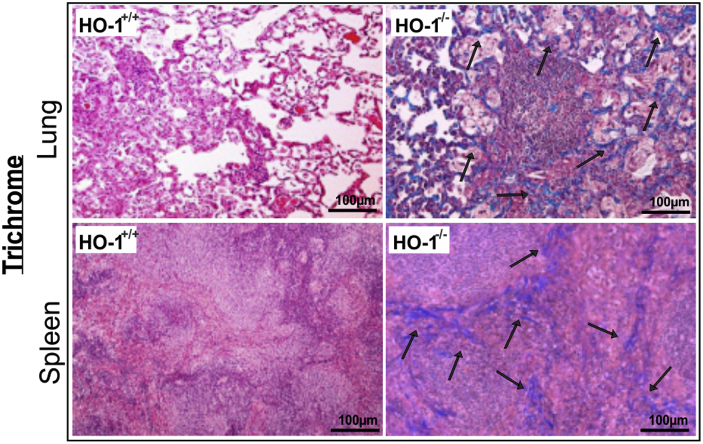
HO-1^−/−^ Mice Are More Susceptible to *Mtb* Infection (original)

