# Supplementary material for: Microanatomic Distribution of Myeloid Heme Oxygenase-1 Protects against Free Radical-Mediated Immunopathology in Human Tuberculosis
Source: Cell Rep. 2018 Nov 13;25(7):1938–1952.e5. doi: 10.1016/j.celrep.2018.10.073 (PMC6250977; doi:10.1016/j.celrep.2018.10.073)
Supplement: Document S1. Figures S1–S7 and Table S1 [file mmc1.pdf]

Cell Reports, Volume *25*

Supplemental Information

Microanatomic Distribution of Myeloid Heme Oxygenase-1 Protects against Free Radical-Mediated Immunopathology in Human Tuberculosis

Krishna C. Chinta, Md. Aejazur Rahman, Vikram Saini, Joel N. Glasgow, Vineel P. Reddy, Jeremie M. Lever, Shepherd Nhamoyebonde, Alasdair Leslie, Ryan M. Wells, Amie Traylor, Rajhmun Madansein, Gene P. Siegal, Veena B. Antony, Jessy Deshane, Gordon Wells, Kievershen Nargan, James F. George, Pratistadevi K. Ramdial, Anupam Agarwal, and Adrie J.C. Steyn

Supplemental Figures.

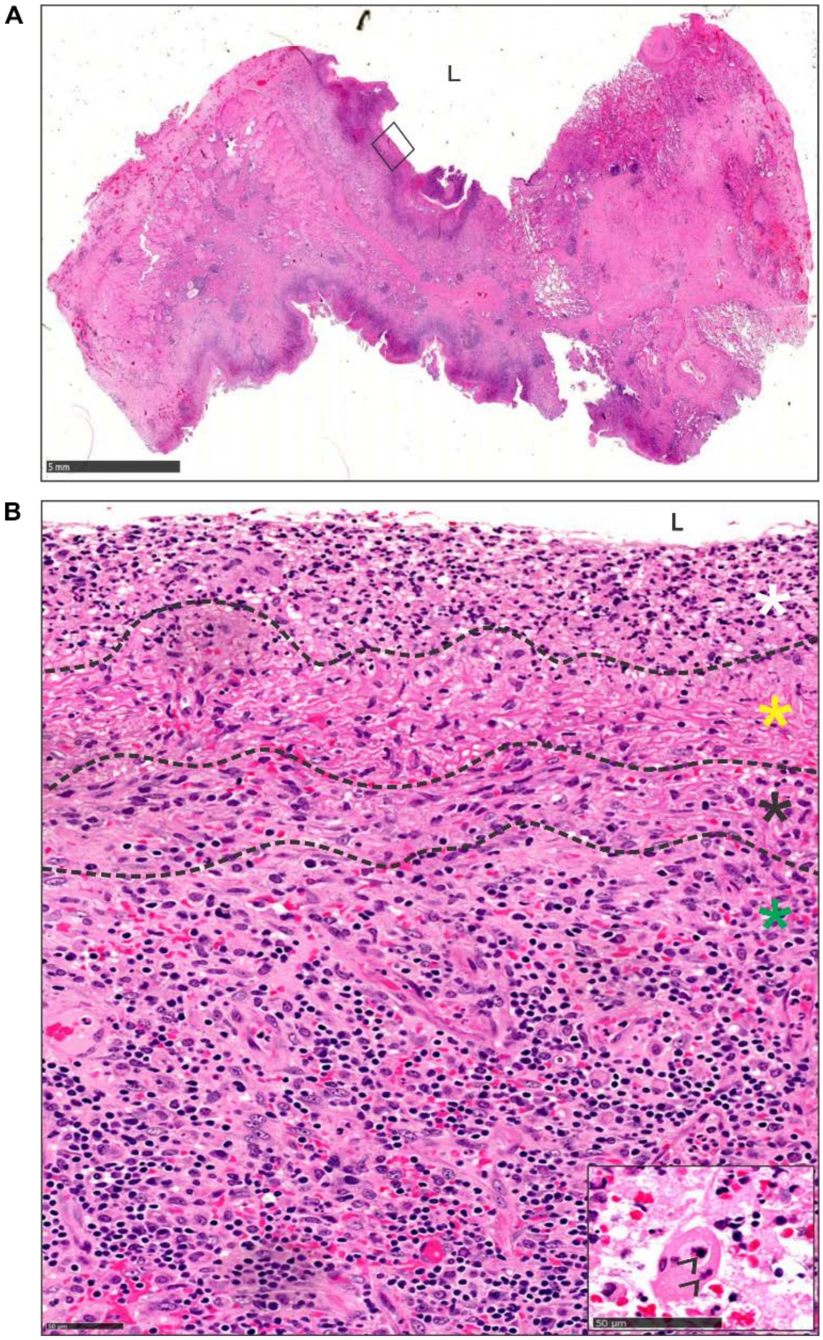

Figure S1. Histomorphology of necrotising granulomatous inflammation: cavity wall. Related to Figure 1. **(A)** Low power H&E demonstration of a tuberculous cavity wall with selected focus (rectangle; Fig. 1) and (**B**) High power spatial demonstration thereof. (B) Adluminal inflammatory cells composed of neutrophils and cellular debris (white asterisk) and focal giant cells (arrow) was juxtaposed to hypocellular fibrinoid necrosis (yellow asterisk). This abutted confluent granulomatous inflammation (black asterisk) composed predominantly of epithelioid histiocytes and scattered lymphocytes. This was bordered by granulation tissue (green asterisk). (L = lumen). Inset: High power demonstration of phagocytic giant cell with intracytoplasmic phagocytized neutrophils (arrowheads).

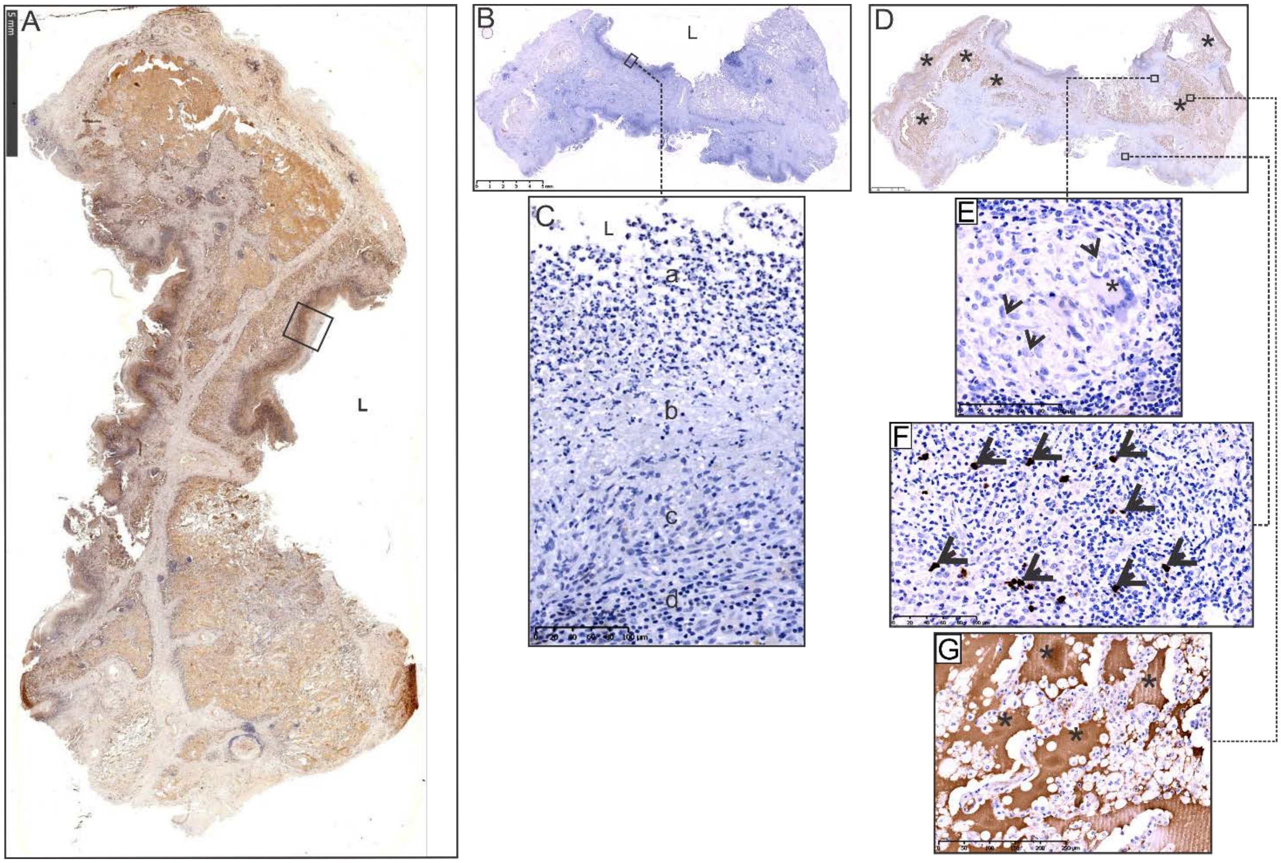

Figure S2. HO-1 staining of cavity wall and isotype control staining in human TB lung tissue. Related to Figure 1 and 2. Low power depiction of (**A**) HO-1 staining of tuberculous cavity wall with selected focus (rectangle) (L = lumen) and **(B**) Antibody control. (**C**) High power depiction of the selected focus highlighting the absence of staining in the wall of the cavity (L = lumen, a, b, c and d represent adluminal suppurative/karyorrhectic, fibrinoid necrosis, granulomatous and granulation tissue zones, respectively). (**D**) IgG4 was employed as the isotype control. Low power whole mount depiction of lgG4 staining in the tissue section. (**E**) High power demonstration of granuloma confirming immunonegative epithelioid histiocytes (black arrows) and a Langhans giant cell (asterisk). Note the lgG4 immunopositive plasma cells (**F**, arrows) in the granulation tissue layer serving as a positive, in-built isotype control response, and surrounding stromal immunonegative endothelial, mesenchymal and lymphoid cells. Additionally, as expected, intra-alveolar blood/serum products demonstrate immunopositivity (**G**, * in C, normal serum lgG4 level is 8-140 mg/dL). In contrast, the viable intra-alveolar and interstitial cellular components were immunonegative. The latter cells have clear cytoplasm and hematoxyphylic (blue) nuclei because of hematoxylin counterstaining.

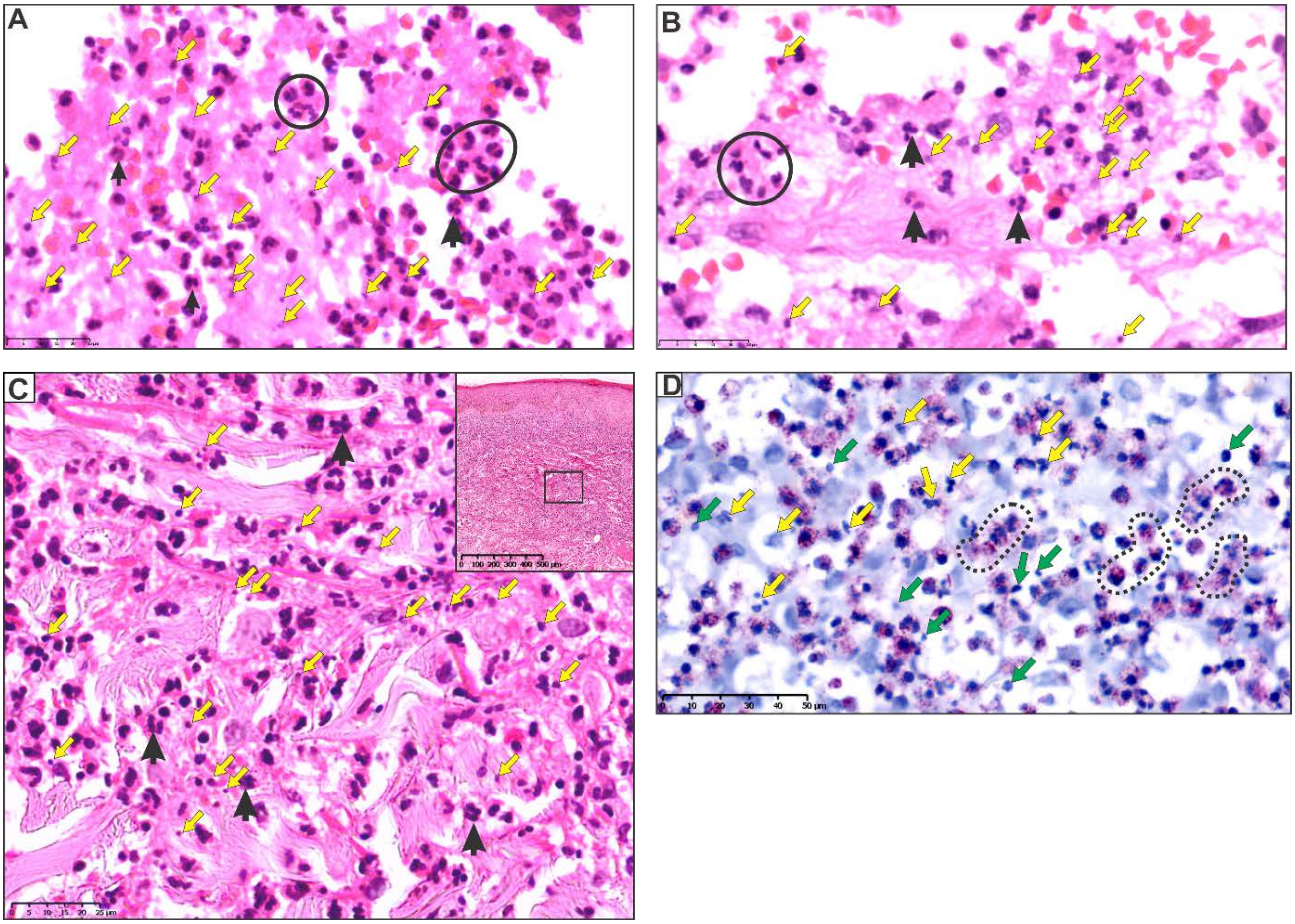

Figure S3. **Karyorrhexis; Histomorphological and histochemical demonstration of neutrophilic degradation. Related to Figure 1 and 2**. Sections of the (**A**) adluminal suppurative/karyorrhectic zone (in FigureS2A) (**B**) abscess contents and (**C**) Sweet syndrome to demonstrate the histomorphological features of karyorrhexis, characterized by nuclear fragmentation. Admixed aggregated (circle) and single (black arrows) intact neutrophils and nuclear (karyorrhectic) nuclear debris in the adluminal layer of the cavity wall. Similar histomorphological features are seen in two other diseases representing positive controls (B, C) in which karyorrhexis is a characteristic histomorphological attribute. Contents of abscess (B) highlighting admixed aggregated (circle) and single (black arrows) intact neutrophils and nuclear debris (karyorrhexis). Low power view of Sweet syndrome (neutrophilic dermatosis) demonstrating a dense cellular dermal infiltrate and high-power demonstration (C) of selected focus (rectangle, C) of intact neutrophils (black arrows) and nuclear fragments (yellow arrows). (**D**) Histochemical demonstration of neutrophil degeneration using chloroacetate esterase [Leder]) stain. Low power demonstration of viable neutrophils containing brightly positive rose-pink granules (dotted line oval shapes), degenerate trinucleate neutrophils (yellow arrows), and nuclear debris (green arrows) lacking chloroacetate esterase positive granules. Degenerate neutrophils lose their enzymatic cellular function, which is highlighted by degranulation and the loss of chloroacetate esterase-stained cytoplasmic granules in tissue sections.

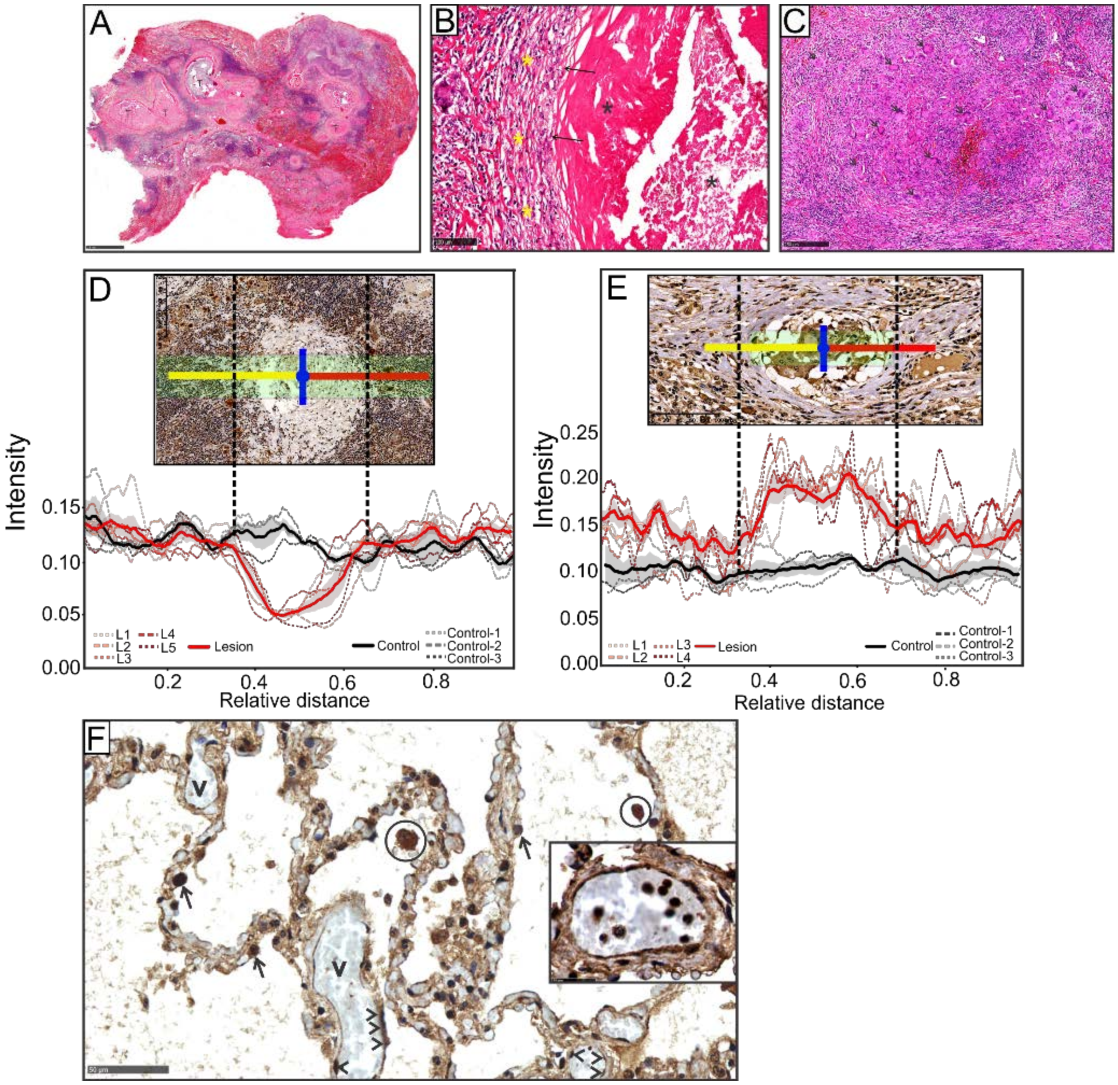

Figure S4, Histomorphology and HO-1 distribution in granulomas and healthy tissues. Related to Figure 2. (**A**) H&E staning of sections of lung parenchyma demonstrating congestion, hemorrhage and tubercles (T) on low power characterized by (**B**) necrotizing granulomatous inflammation with central caseative necrosis (black asterisks) surrounded by a fibro-inflammatory response consisting of granulomas (arrows) and vascularized lamellar fibrosis (yellow asterisks) on high power. (**C**) Medium power H&E depiction of confluent organized granulomas composed of Langhans giant cells (arrows), epithelioid histiocytes and lymphocytes. Decreased HO-1 levels within caseous necrotic lesions (**D**) and increased HO-1 levels in non-necrotic granulomatous inflammatory (NNGI) lesions (**E**). Representative images corresponding to caseous necrotic and NNGI are included for purposes of clarity; the scale does not correspond to the relative distance scale in the graphs. DAB stain intensity relative to a masked region of fixed size is plotted for five individual lesions (L) (dotted/dashed red series) and three control regions (dotted/dashed grey series) (**D**). Four lesions and three control regions were examined in **E**. Combined plots for granulomatous (red) and control (black) are indicated with confidence bands (shaded regions) at a confidence interval of 0.68. (**F**) HO-1 positive alveolar pneumocytes (arrows), endothelial cells in interstitial vessels (arrowheads) and focal intra-alveolar histiocytes (circles). Inset: HO-1 positive circulating intravascular neutrophils. V = vessel (blood).

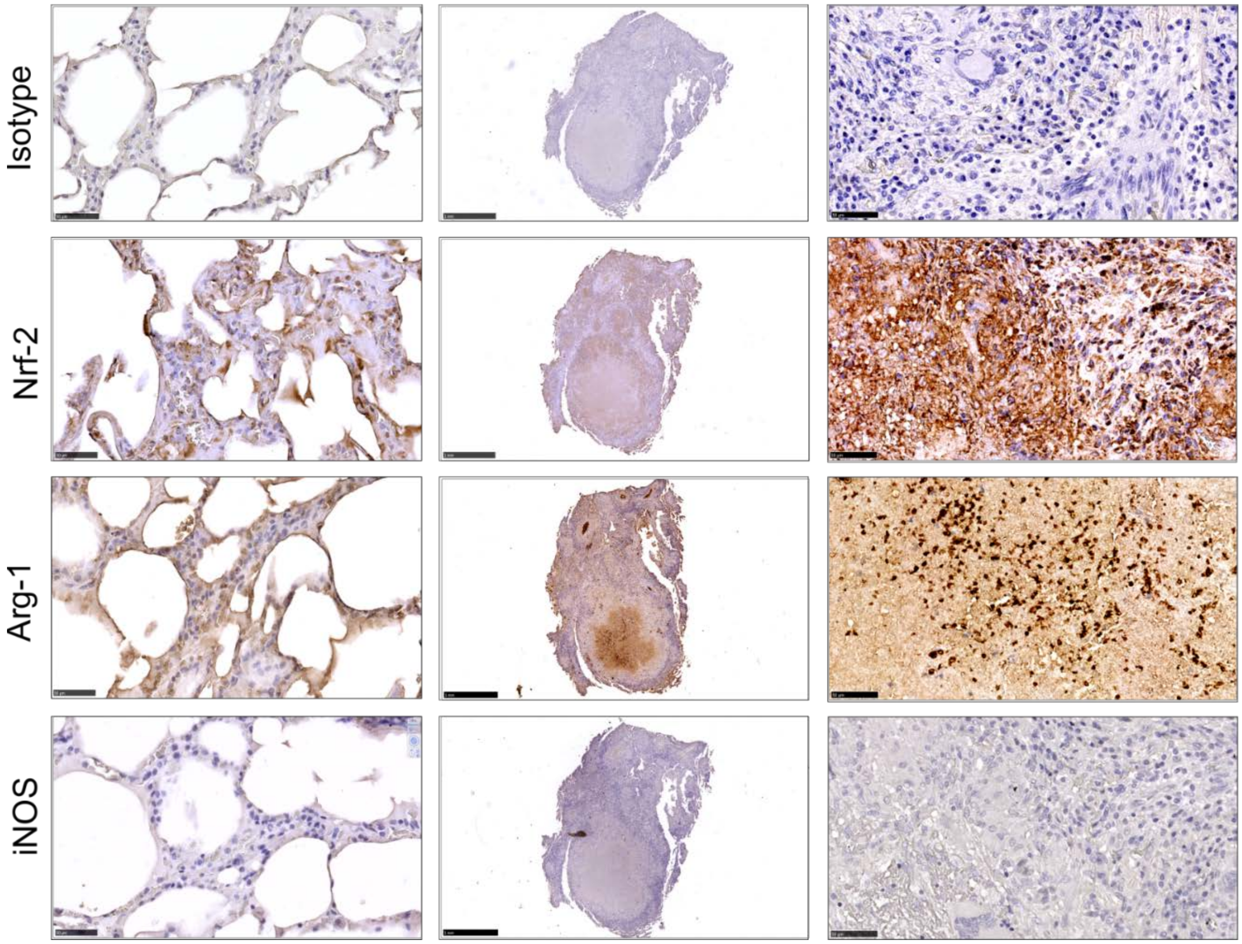

Figure S5. Nrf-2, iNOS and Arg-1 are produced in human TB lung lesions, Related to Figure 2. Representative tissue sections showing immunohistological detection of Nrf-2, Arg-1 and iNOS levels in diseased and healthy lung regions of TB patients (Images are represented at either 2x or 40x magnification). An immunonegative rabbit IgG isotype control is shown (isotype).

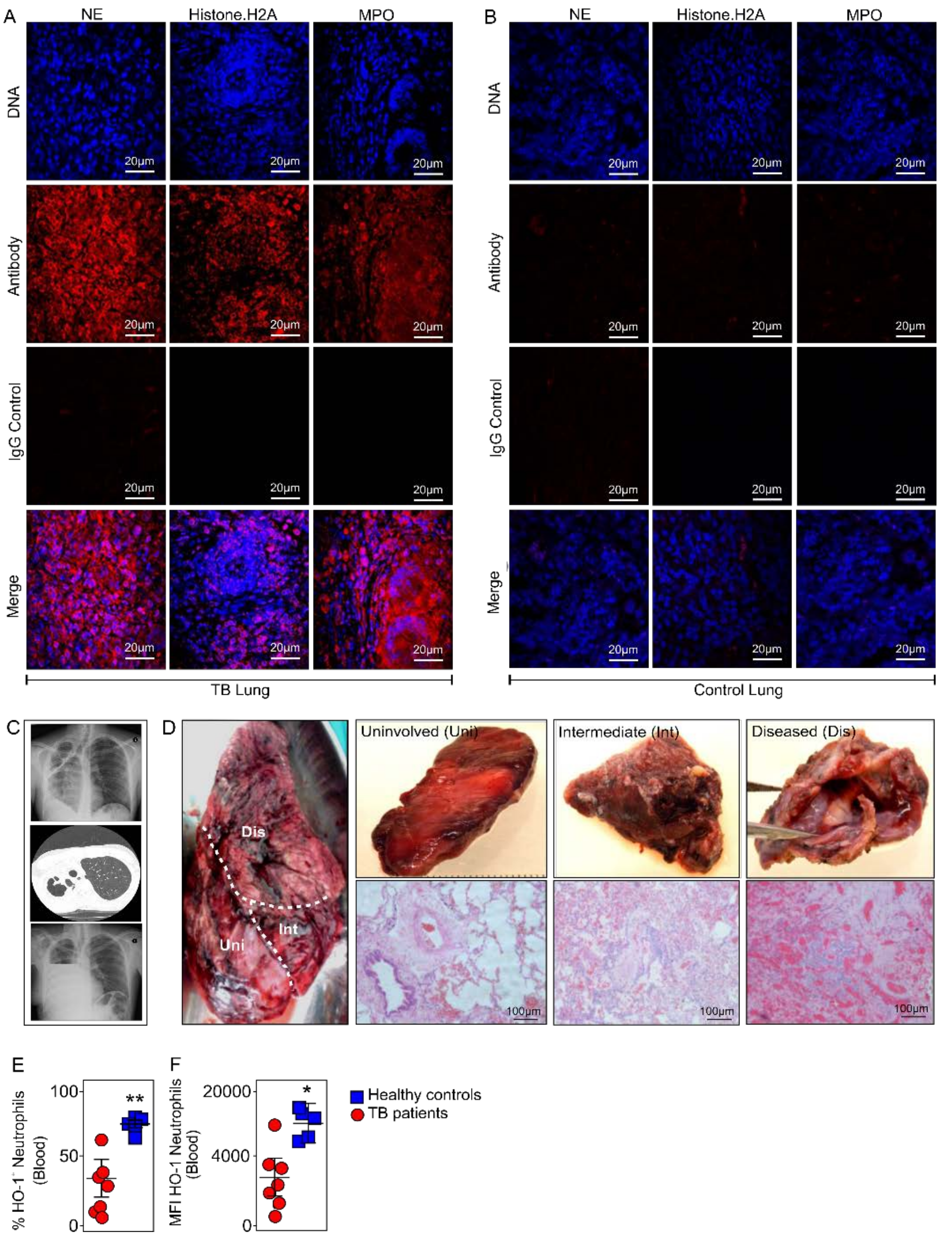

Figure S6. Distinct pathological regions, neutrophil accumulation in human TB lungs and HO-1 levels in the blood of TB patients. Related to Figure 3. Representative lung tissue sections showing immune-fluorescence detection of MPO, neutrophil elastase and histone.H2A which are markers of NETs in (**A**) TB patients and (**B**) control non-TB lung. (Images taken at 40x magnification). (**C**) Representative chest radiograph showing a shrunken right lung with a large cavity in the right upper lobe (top). The same patient underwent high-resolution computed tomography (HRCT) scan, which confirmed the cavity in the right upper lobe (middle). Second chest radiograph of same patient, post-pneumonectomy (bottom). (**D**) Resected lung of same patient and identification of distinct pathological regions by gross pathology and H&E staining (Images taken at 10x magnification). (**E**, **F**) HO-1 levels in the neutrophils ($CD11b^+CD66b^+CD16^+CD14^-CD3^-$) isolated from the blood of TB patients compared to healthy controls. (**E**) Percent HO-1^+ neutrophils in the blood of Tb patients compared to healthy controls (**F**) MFI of HO-1 in neutrophils.

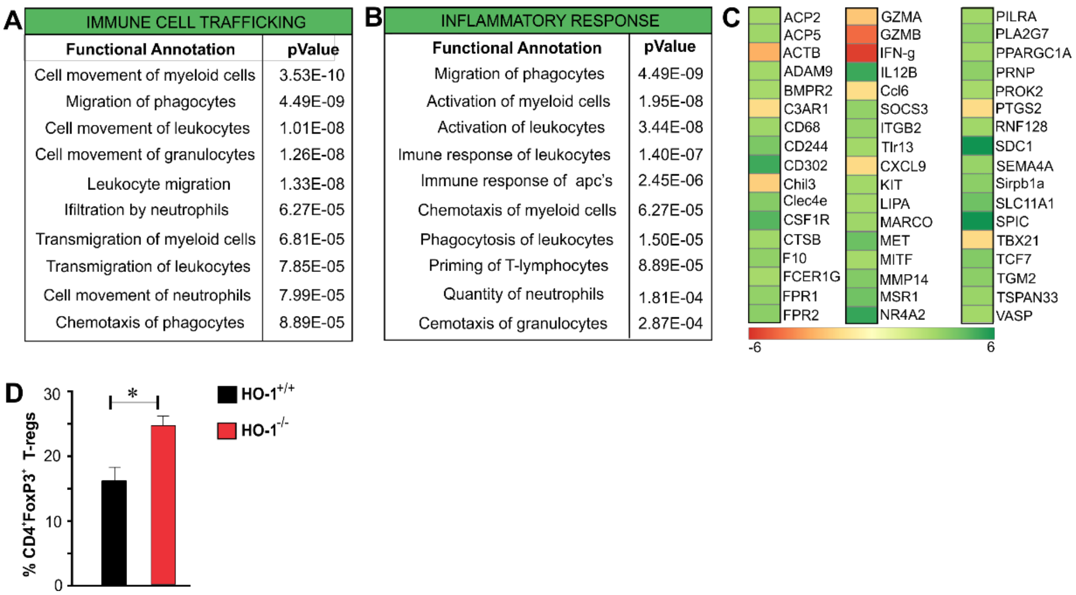

Figure S7. Global transcriptomic profiles of blood monocytes from HO-1$^{+/+}$ and HO-1$^{-/-}$ mice following *Mtb* infection and increased T-reg accumulation in HO-1$^{-/-}$ mice. **Related to Figure 5**. Ingenuity Pathways Analysis (IPA) pathway analysis showing the most significantly ranked pathways in the categories of (**A**) immune cell trafficking and (**B**) inflammatory responses. The results are ordered by -log10 of the *p*-value of the hypergeometric distribution. (**C**) Significantly regulated genes in the pathways mentioned in the A and B. Fold change greater than 1.5 was considered as significant. (**D**) Percent differences in CD4^{+}FoxP3^{+} T-regs in the lungs of *Mtb*-infected HO-1$^{+/+}$ and HO-1$^{-/-}$ mice at 18 weeks post infection. n = 4 for each experiemnt. Statistical testing was performed using the unpaired Student's t-test. Data are represented as mean ± SEM. *p < 0.05.

Supplemental Table.

ID	Age	Sex	TB Type	Type of Resection	Drug treated
32	27	Female	TB	LLP	Yes
104	33	Female	TB	RUL	Yes
110	34	Female	MDR TB	RLP	Yes
30	39	Female	TB	RUL	Yes
23	40	Female	XDR TB	RLP	Yes
26	41	Female	MDR-TB	LLP	Yes
25	42	Female	MDR-TB	LLP	Yes
114	44	Female	TB	LLP	Yes
42	46	Female	TB	RLP	Yes
24	21	Male	MDR-TB	RLP	Yes
40	26	Male	MDR TB	LUL	Yes
37	27	Male	TB	LUL	Yes
27	31	Male	TB	RLP	Yes
107	33	Male	TB	LUL	Yes
115	35	Male	TB	LUL	Yes
103	39	Male	TB	LLP	Yes
102	40	Male	TB	LLP	Yes
38	41	Male	MDR TB	RLP	Yes
113	52	Male	TB	LUL	Yes
108	56	Male	TB	LWL	Yes
31	66	Male	TB	RUL	Yes

Table S1. Clinical characteristics of human subjects. Related to Figure 1, 2,3 and Star Methods. Males and female patients were recruited at King DinuZulu Hospital Complex, Durban, South Africa. Written consent was taken from all the patients. Before recruitment all the patients were subjected to tests described in Supplemental Experimental Procedures. Acronyms used: TB. Tuberculosis; LLP. Left Lung Pneumonectomy; RLP. Right Lung Pneumonectomy; RUL. Right Upper Lobectomy; LUL. Left Upper Lobectomy; LWL. Left Whole Lobectomy; MDR-TB. Multi Drug-Resistant TB; XDR-TB. Extensively Drug-Resistant TB.
