# Supplementary material for: Microanatomic Distribution of Myeloid Heme Oxygenase-1 Protects against Free Radical-Mediated Immunopathology in Human Tuberculosis
Source: Cell Rep. 2018 Nov 13;25(7):1938–1952.e5. doi: 10.1016/j.celrep.2018.10.073 (PMC6250977; doi:10.1016/j.celrep.2018.10.073)
Supplement: Document S2. Article plus Supplemental Information [file mmc3.pdf]

Cell Reports

Article

# Microanatomic Distribution of Myeloid Heme Oxygenase-1 Protects against Free Radical-Mediated Immunopathology in Human Tuberculosis

## Graphical Abstract

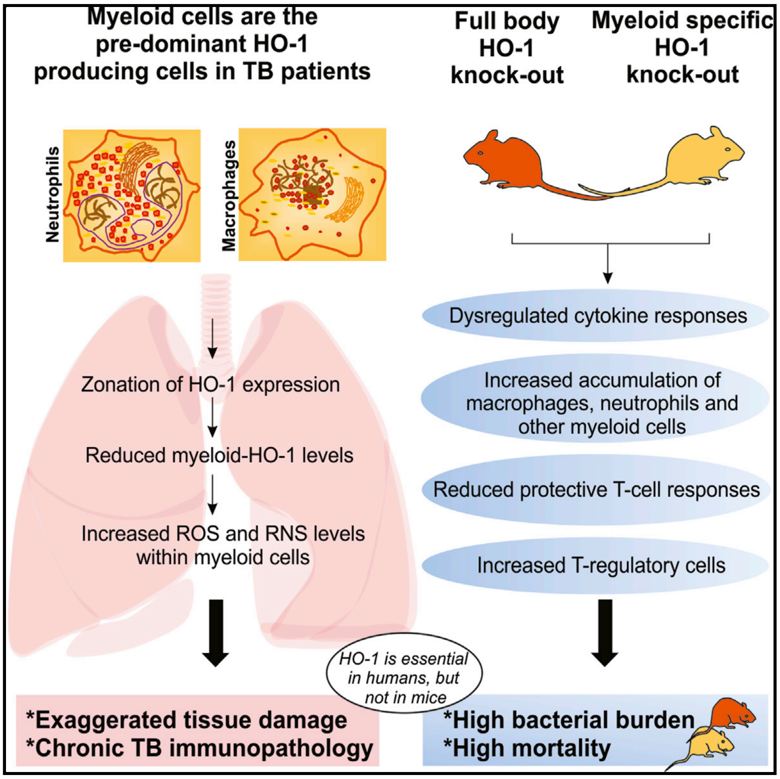


## Highlights

- HO-1 is expressed in distinct microanatomic zones within the human TB lung
- Macrophages and neutrophils are the major producers of HO-1 in human TB lungs
- In diseased areas of human TB lungs, myeloid cells have low HO-1 and high ROS and RNS
- Mice lacking HO-1 expression in myeloid cells are more susceptible to *Mtb* infection

## Authors

Krishna C. Chinta, Md. Aejazur Rahman, Vikram Saini, ..., Pratistadevi K. Ramdial, Anupam Agarwal, Adrie J.C. Steyn

## Correspondence

asteyn@uab.edu

## In Brief

Heme oxygenase-1 (HO-1) is a cytoprotective enzyme that controls inflammation and redox homeostasis; however, its role in tuberculosis (TB) is unclear. Using freshly resected human lung tissue and HO-1-deficient mice, Chinta et al. demonstrate that HO-1 in myeloid cells is important for controlling inflammatory and free-radical-mediated tissue damage in TB.

Chinta et al., 2018, Cell Reports *25*, 1938–1952
November 13, 2018 © 2018 The Author(s).
https://doi.org/10.1016/j.celrep.2018.10.073

CellPress

OPEN
ACCESS
CellPress

Cell Reports
Article

# Microanatomic Distribution of Myeloid Heme Oxygenase-1 Protects against Free Radical-Mediated Immunopathology in Human Tuberculosis

Krishna C. Chinta,[1] Md. Aejazur Rahman,[2] Vikram Saini,[1] Joel N. Glasgow,[1] Vineel P. Reddy,[1] Jeremie M. Lever,[4] Shepherd Nhamoyebonde,[2] Alasdair Leslie,[2] Ryan M. Wells,[1] Amie Traylor,[4] Rajhmun Madansein,[5] Gene P. Siegal,[6] Veena B. Antony,[7] Jessy Deshane,[7] Gordon Wells,[2] Kievershen Nargan,[2] James F. George,[3] Pratistadevi K. Ramdial,[8] Anupam Agarwal,[4,9] and Adrie J.C. Steyn[1,2,10,11,12,*]

[1]Department of Microbiology, School of Medicine, The University of Alabama at Birmingham, Birmingham, AL 35294, USA
[2]Africa Health Research Institute, Durban 4001, South Africa
[3]Division of Cardiothoracic Surgery, Department of Surgery, The University of Alabama at Birmingham, Birmingham, AL 35294, USA
[4]Nephrology Research and Training Center, Division of Nephrology, The University of Alabama at Birmingham, Birmingham, AL 35294, USA
[5]Inkosi Albert Luthuli Central Hospital, Durban 4041, South Africa
[6]Department of Pathology, The University of Alabama at Birmingham, Birmingham, AL 35294, USA
[7]Division of Pulmonary, Allergy and Critical Care Medicine, Department of Medicine, The University of Alabama at Birmingham, Birmingham, AL 35294, USA
[8]Department of Anatomical Pathology, NHLS, Inkosi Albert Luthuli Central Hospital, University of KwaZulu-Natal, Durban 4091, South Africa
[9]Department of Veterans Affairs, Birmingham, AL 35294, USA
[10]UAB Center for AIDS Research, The University of Alabama at Birmingham, Birmingham, AL 35294, USA
[11]Center for Free Radical Biology, The University of Alabama at Birmingham, Birmingham, AL 35294, USA
[12]Lead Contact
*Correspondence: asteyn@uab.edu
https://doi.org/10.1016/j.celrep.2018.10.073

## SUMMARY

**Heme oxygenase-1 (HO-1) is a cytoprotective enzyme that controls inflammatory responses and redox homeostasis; however, its role during pulmonary tuberculosis (TB) remains unclear. Using freshly resected human TB lung tissue, we examined the role of HO-1 within the cellular and pathological spectrum of TB. Flow cytometry and histopathological analysis of human TB lung tissues showed that HO-1 is expressed primarily in myeloid cells and that HO-1 levels in these cells were directly proportional to cytoprotection. HO-1 mitigates TB pathophysiology by diminishing myeloid cell-mediated oxidative damage caused by reactive oxygen and/or nitrogen intermediates, which control granulocytic karyorrhexis to generate a zonal HO-1 response. Using whole-body or myeloid-specific HO-1-deficient mice, we demonstrate that HO-1 is required to control myeloid cell infiltration and inflammation to protect against TB progression. Overall, this study reveals that zonation of HO-1 in myeloid cells modulates free-radical-mediated stress, which regulates human TB immunopathology.**

## INTRODUCTION

*Mycobacterium tuberculosis* (*Mtb*), the etiological agent of tuberculosis (TB) disease, is a highly contagious pathogen that is spread via inhalation of infectious droplets released from the lungs of infected individuals. While the disease is mostly asymptomatic, active pulmonary TB, characterized by extensive hemoptysis and extensive tissue damage, is often lethal. The formation of caseous granulomas is a hallmark of *Mtb* infection that involves accumulation of myeloid cells, including neutrophils, macrophages, and myeloid-derived suppressor cells (MDSCs) to infected sites (du Plessis et al., 2013; Obregón-Henao et al., 2013; Silva Miranda et al., 2012). While the formation of granulomas can be beneficial for the host, it may also serve as a safe niche for the bacterium (Silva Miranda et al., 2012). In addition, the uncontrolled infiltration of myeloid cells and subsequent inflammation, including reactive oxygen intermediates (ROIs) and reactive nitrogen intermediates (RNIs), may contribute to disease pathology (Chinta et al., 2016). However, most studies on myeloid-mediated inflammation in TB have relied on animal models or blood from TB patients. Assessing the clinical relevance of these findings is difficult, because animal models and *ex vivo* blood analysis have limitations in recapitulating human disease (Dharmadhikari and Nardell, 2008). Further, the microanatomic architecture of human pulmonary TB is mostly unexplored, largely due to the paucity of resected human tuberculous lung tissue. Not surprisingly, correlating the immune state of the patient and the clinicopathological manifestations of pulmonary TB lesions has been difficult, as is evident by few reports dated decades ago (Lenzini et al., 1977; Ridley and Ridley, 1987).

Heme oxygenase-1 (HO-1) is a redox-sensitive cytoprotective enzyme that degrades heme, a potent oxidant, to yield equimolar ratios of carbon monoxide (CO), iron, and bilirubin (Tenhunen et al., 1968). HO-1 protects cells from heme-mediated oxidative and nitrosative stress and injury and is involved in myeloid cell

 © 2018 The Author(s).
This is an open access article under the CC BY license (http://creativecommons.org/licenses/by/4.0/).

recruitment and T cell responses in many pathological conditions (Castilho et al., 2012; Choi and Alam, 1996; Freitas et al., 2006; George et al., 2008). We and others have shown that HO-1 is upregulated in response to *Mtb* infection in mice and responds independently of the interferon-$\gamma$ (IFN-$\gamma$)/nitric oxide (NO) pathway and that HO-1-generated CO is required for the induction of the *Mtb* Dos dormancy regulon (Kumar et al., 2008; Shiloh et al., 2008). HO-1 is required to control *Mycobacterium avium* and *Mtb* infections in mice (Regev et al., 2012; Silva-Gomes et al., 2013). In addition, it was recently shown that the free heme iron released by HO-1 enzymatic activity is bound by ferritin H, which is required to control *Mtb* infection in mice (Reddy et al., 2018). Also, HO-1 levels in the plasma of TB can distinguish patients with active TB from latently infected individuals (Andrade et al., 2013), as a readout for the efficacy of TB therapy or diagnosis of TB-HIV co-infection (Rockwood et al., 2017). Furthermore, HO-1 levels in plasma were reported to be inversely correlated with the levels of matrix metalloproteinases, which contribute to tissue destruction in TB (Andrade et al., 2015; Salgame, 2011). More recently, studies have challenged the beneficial role of HO-1 in TB disease, reporting that pharmacological inhibition of HO-1 in mice leads to a decrease in *Mtb* burden (Costa et al., 2016; Scharn et al., 2016). These conflicting findings, in addition to the fact that the essentiality of HO-1 in humans and mice varies significantly, represent a substantial gap in our understanding of the role of HO-1 in TB.

In this study, we tested the hypothesis that HO-1 is essential for effective immune and oxidative stress control to limit TB pathology in mice and human tuberculous lungs. To test this hypothesis, we used multiparameter flow cytometry and immunohistochemistry to examine HO-1 expression in freshly resected and fixed lung tissues of TB patients. The spatial distribution of HO-1 within the microenvironment of human pulmonary TB lesions was also examined. Using global HO-1 knockout (HO-1$^{-/-}$) and myeloid cell-specific HO-1 knockout (HO-1$^{LysM-/-}$) mice, we studied the survival, disease progression, transcriptional changes, and immune responses upon *Mtb* infection. Overall, our data show that the expression of HO-1, especially within myeloid cells, is essential for host defense against TB disease.

## RESULTS

### Cellular Distribution of HO-1 within the Histopathological Spectrum of TB

Historically, clinical and immunological studies have attempted to define the clinicopathological manifestations of TB disease and relate them to the immune state of TB patients. However, a correlation between the immune state and the pathological spectrum is lacking (Barry et al., 2009; Ridley and Ridley, 1987). To determine the role of HO-1 within the pathological spectrum of TB, we examined the microanatomic distribution of HO-1 within human TB lungs. Pathologic features were appraised in terms of necrotizing (cavity wall, tubercle), non-necrotizing granulomas, and control lung sections.

#### *Cavity Wall*

Microscopically, the lumen contained erythrocytes, an adluminal exudative component composed mainly of neutrophils, nuclear debris, and giant cells, including phagocytic giant cells (Figures S1A and S1B). Fibrinoid necrosis was noted, in addition to a confluent granulomatous layer composed mainly of epithelioid histiocytes, some of which demonstrated palisading and outermost inflamed granulation tissue (Figure S1B). HO-1 staining of different cavity wall components was variable (Figure S2A). HO-1 staining was bright in giant cells (Figure 1, inset i), the granulomatous inflammatory component, and endothelial cells in all layers, but it was especially bright in the granulation tissue (Figure 1). Negative controls using a secondary antibody alone (Figures S2B and S2C) or an isotype control antibody (Figures S2D–S2G) showed immunonegative reactions, demonstrating the specificity of HO-1 staining. Intact neutrophils, lymphocytes, histiocytes, and plasma cells stained brightly for HO-1 (Figure 1, inset ii). However, karyorrhectic (leukocytoclastic) neutrophils and nuclear debris were unstained (Figure 1, inset iii). Karyorrhexis is a histomorphological, usually diagnostic feature present in a variety of diseases, including infective (bacillary angiomatosis) (Laga and Milner, 2015) and non-infective sources (Barksdale et al., 2015) (e.g., Wegener granulomatosis, erythema elevatum diutinum, granuloma faciale, small-vessel neutrophilic and/or leucocytoclastic vasculitis, and cryoglobulinemia). Further evidence supporting karyorrhexis was obtained through histomorphology and histochemical features in the adluminal suppurative and/or karyorrhectic zone in TB lung tissue (Figure S3A). Positive controls include an abscess highlighting intact neutrophils and nuclear karyorrhectic debris (Figure S3B) and a Sweet syndrome section (neutrophil dermatosis) showing a dense cellular dermal infiltrate of intact neutrophils and neutrophil karyorrhexis (Figure S3C). Both controls show clear histomorphological features consistent with karyorrhexis in TB lung tissue. Lastly, to address the relationship between karyorrhexis and dying cells histologically, we performed chloroacetate esterase (CAE) staining routinely used for identification of neutrophil degeneration and hematologic disorders. This method of identifying neutrophil degeneration in tissue sections is highlighted by degranulation and the loss of chloroacetate-esterase-stained (rose-pink) cytoplasmic granules (Figure S3D).

#### *Tubercles*

Tubercles were identified at low magnification by the central necrosis (Figures S4A and S4B). Higher magnification revealed that the central necrotic region was surrounded by a fibro-inflammatory and granulomatous region (Figure S4B). Within the microanatomic components of the tubercles, some neutrophils in the caseative component, giant cells, and histiocytes in granulomas and endothelial cells in the outer vascularized fibrous lamellae demonstrated high levels of HO-1 protein (Figures 2A–2D). The granular caseative debris was HO-1 negative (Figure 2B).

#### *Non-necrotizing Granulomatous Inflammation*

The granulomas that were variably aggregated had an organized morphology and were composed of Langhans giant cells, histiocytes, and lymphocytes (Figure S4C). HO-1 staining in the granulomas within the non-necrotizing granulomatous inflammation (NNGI) foci was similar to that in the tubercles and cavity wall (Figures 2C and 2D). A morphometric appraisal of necrotic and NNGI lesions demonstrated striking differences in the spatial

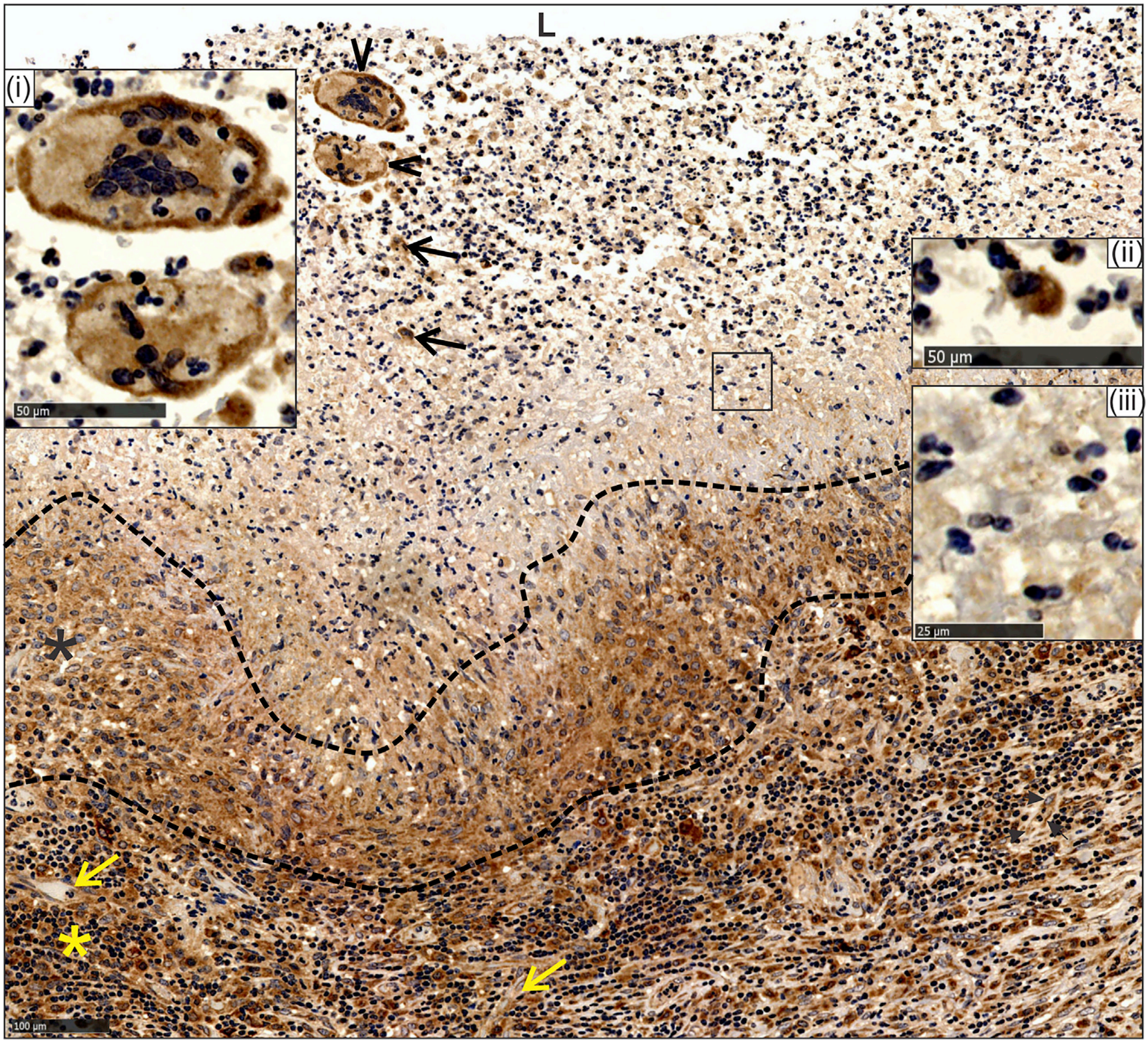


**Figure 1. HO-1 Staining Profile in the Cavity Wall**

HO-1 staining of cellular component in adluminal cells with bright staining of phagocytic giant cells (arrowheads, inset i), bright staining of neutrophils (arrows, inset ii), and negative karyorrhectic neutrophil staining (rectangle, inset iii) (L = Lumen). Shown is bright staining of histiocytes in the granulomatous layer (black asterisks) and bright staining of inflammatory cells (lymphocytes, plasma cells, and histiocytes) and endothelial cells (yellow arrows) in the granulation tissue layer (asterisk).

distribution of HO-1. In necrotic lesions, HO-1 levels were significantly reduced, whereas in NNGI lesions, HO-1 levels increased (Figures S4D and S4E). Healthy lung control sections demonstrated normal alveolar spaces, septa, and vascular and bronchiolar components (Figure S4F). In the control sections, the aerated compartments, including pneumocytes and intra-alveolar histiocytes, were strongly HO-1 positive. In addition, cells in oxygen-rich tissue such as endothelial cells within capillaries and circulating cells also expressed high levels of HO-1. Scattered intra-alveolar histiocytes were present. HO-1 staining was observed in pneumocytes, endothelial cells in the interstitial vessels, focal intra-alveolar histiocytes, and circulating intravascular neutrophils (Figure S4F, inset).

In summary, the histopathological appraisal of TB lung specimens revealed a defined spectrum of microscopic abnormalities, including hemorrhage. The spatial distribution of HO-1 in different tuberculous lesions reflects different architectural and cellular patterned responses. Notably, while Langhans giant cells, histiocytes, plasma cells, and endothelial cells in the microanatomic locations stained positively for HO-1, karyorrhectic

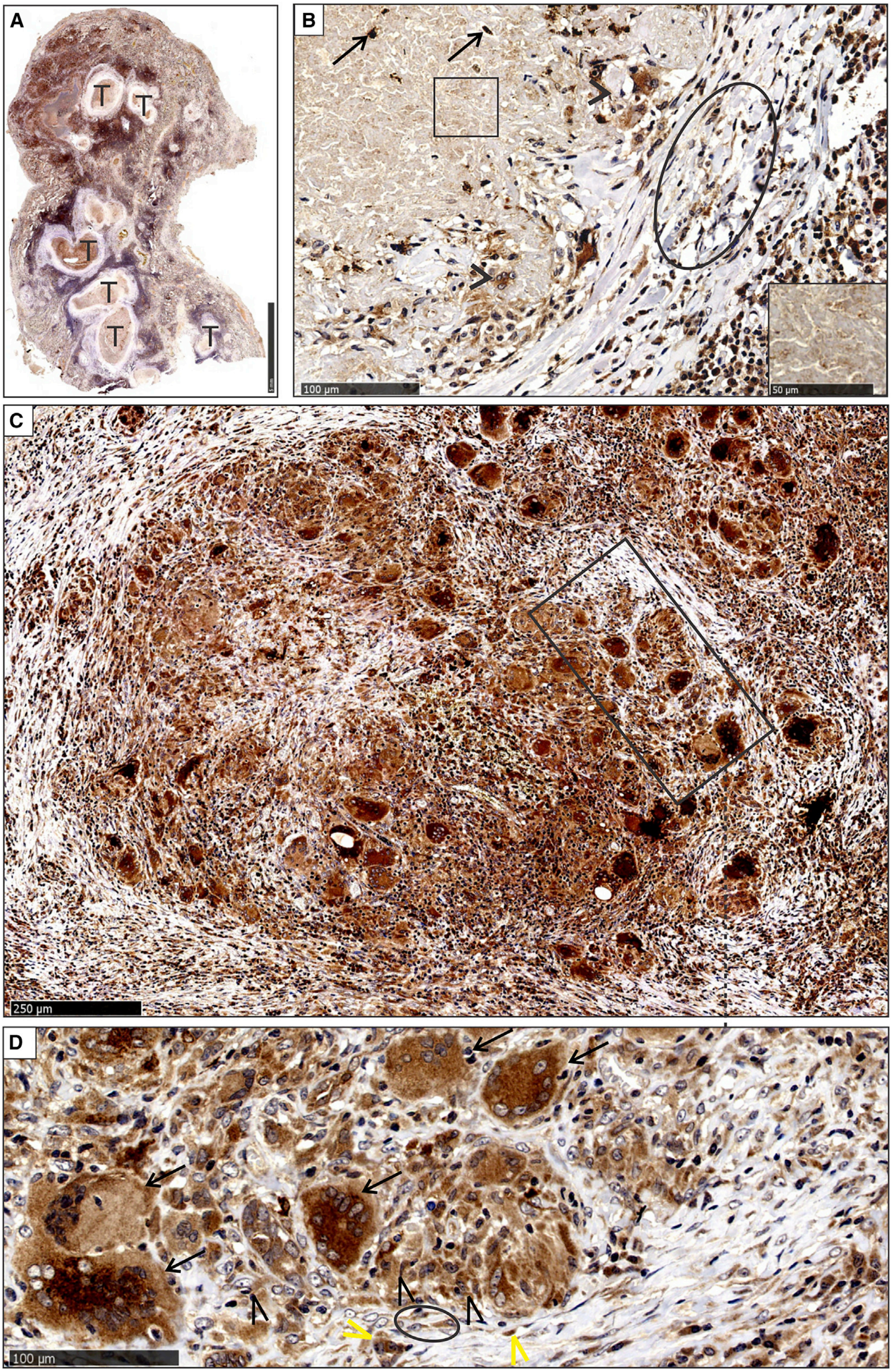


(legend on next page)

neutrophils consistently showed markedly reduced HO-1 levels, particularly in cavities and near necrotic granulomatous foci. Notably, the cell-type-specific expression of HO-1 within discrete microanatomical locations, which generates a zonal distribution, together with the essential role HO-1 plays in human health, provides evidence for a central role of HO-1 in the pathophysiology of TB.

## Arg-1, Nrf2, iNOS, and Neutrophil Levels in the Lungs of TB Patients

Immunohistochemical (IHC) analysis revealed that expression of Nrf2, a major transactivator of HO-1 promoter activity, was increased in human TB lungs compared to non-TB controls (Figure S5). Considering HO-1 downstream signaling is known to modulate expression of Arginase-1 (Arg-1) and inducible nitric oxide synthase (iNOS) (Bolisetty et al., 2015; Datta et al., 1999), we used IHC to map these proteins in human lung tissue. We observed increased Arg-1 staining in TB diseased tissue, which is consistent with other studies (Duque-Correa et al., 2014), but found no measurable differences in iNOS levels between TB diseased and control tissues (Figure S5). No staining was observed in isotype control images. Overall, our results demonstrate that Nrf2, iNOS, and Arg-1 are produced at the site of infection, suggesting a role for them in human TB. Histopathological examination revealed extensive karyorrhexis, an oxidative-stress-induced degenerative process that releases DNA. Therefore, we examined whether neutrophil extracellular traps (NETs) are formed in human TB lungs using standard markers neutrophil elastase (NE) and myeloperoxidase (MPO). Indeed, significant accumulation of neutrophils in human TB lung tissue was observed (Figure S6A), but not in control lung (Figure S6B). Positive histone H2A staining near NE- and MPO-positive neutrophils strongly suggest the formation of NETs in the human tuberculous lung.

## HO-1 Levels in Myeloid Cells from Pathologically Distinct Regions of Human TB Lungs

Distinct immune cell populations in the blood, pleural cavity fluid, and bronchoalveolar lavage fluid (BALF) of TB patients have been described. However, to the best of our knowledge, there has been no detailed flow cytometric characterization of immune cell populations within the human TB lung. Here we examined the cellular distribution of HO-1 in freshly resected lung tissue from 21 TB patients (Table S1). A representative chest radiograph of a patient with advanced TB prior to pneumonectomy demonstrates a shrunken right lung with a large cavity in the upper lobe, which was then confirmed by a high-resolution CT scan (Figure S6C). The post-pneumonectomy chest radiograph is also shown. The resected lung exhibited distinct regions with varying disease severity that include highly diseased (Dis), intermediate disease (Int), and uninvolved (Uni) areas with normal lung architecture (Figure S6D). As reported in cancer patients (Sellers et al., 2015), examination of lung tissues with varying disease severity from the same patient eliminates potentially confounding intrinsic genetic variables and is optimal for analysis of host responses during TB disease progression.

$CD45^+$ leukocytes were isolated from pathologically distinct regions (diseased, intermediate, and uninvolved) within human TB lungs. From these $CD45^+$ populations, we sorted neutrophils ($CD11b^+CD66b^+CD16^+CD14^-CD3^-$), macrophages ($HLA^-DR^+ CD11c^+CD206^+CD11b^+CD86^+CD16^-CD66b^-CD3^-$), monocytes ($CD14^+HLA\text{-}DR^+CD11c^{low}CD66b^-CD3^-$), and T cells ($CD3^+CD14^-CD11b^-$) and determined the intracellular HO-1 levels in these cell types (Figure 3A). Neutrophils and macrophages were the predominant HO-1-producing cell types. Surprisingly, the percentages of HO-1-producing neutrophils, macrophages, and monocytes isolated from diseased regions were significantly reduced compared to cells isolated from uninvolved regions (Figures 3B–3J). More importantly, we observed that the mean fluorescent intensity (MFI) of HO-1 immunostaining was reduced in neutrophils, macrophages, and monocytes isolated from diseased lung regions compared to intermediate and healthy uninvolved regions (Figures 3B–3J). T cells produced very low or no HO-1 (data not shown). Interestingly, a comparison of the relative HO-1 levels within the same four cell populations isolated from the blood of TB patients showed that compared to healthy individuals, HO-1 levels are reduced in circulating neutrophils of TB patients (Figures S6E and S6F). HO-1 was low or undetectable in all other leukocyte types isolated from the blood (data not shown), suggesting that the immune cell composition of the blood may not accurately reflect the microenvironment of the human tuberculous lung.

Overall, we show that macrophages and neutrophils are the predominant HO-1-producing immune cells in the human TB lung and that reduced HO-1 levels in these cells correspond with increased disease severity. The flow cytometry findings are consistent with our histopathological data showing that the most diseased foci (cavitary and necrotic granulomatous lesions) contain karyorrhectic neutrophils with reduced HO-1 staining.

## Myeloid Cells with Decreased HO-1 Exhibit Increased ROS and Reactive Nitrogen Species Production

Given that HO-1 has potent antioxidant activity, reduced levels of HO-1 in cavitary, coagulative, and necrotic granulomatous foci of human TB lungs indicate that these regions may be exposed to high levels of oxidative stress. Therefore, we measured ROIs and RNIs in the same immune cell isolates in which we measured HO-1 production. Using the superoxide ($O_2^{\bullet -}$)-reactive dye dihydroethidium (DHE), we observed significant increases in the percentages of reactive oxygen species (ROS)-positive neutrophils, macrophages, and monocytes

**Figure 2. HO-1 Staining of Tubercles and Non-necrotizing Granulomas**

(A and B) Low-power magnification of HO-1 staining in lung parenchyma with multiple tubercles (T) (A) and high-power demonstration of HO-1 positivity in scattered neutrophils (arrows) in the central caseative component (B). Also shown are HO-1-negative granular debris (square, inset), HO-1-positive giant cells, and epithelioid histiocytes in granulomas (arrowheads) and HO-1-positive endothelial cells lining capillaries in fibrous lamellae (oval).

(C and D) Bright HO-1 staining of granulomas (rectangle) at medium (C) and high magnification (D). Langhans giant cells (D, arrows) and epithelioid histiocytes (D, black arrowheads) were strongly HO-1 positive. HO-1-positive endothelial cells (yellow arrowheads) in the adjacent vasculature and myofibroblasts (oval) are shown.

OPEN
ACCESS
CellPress

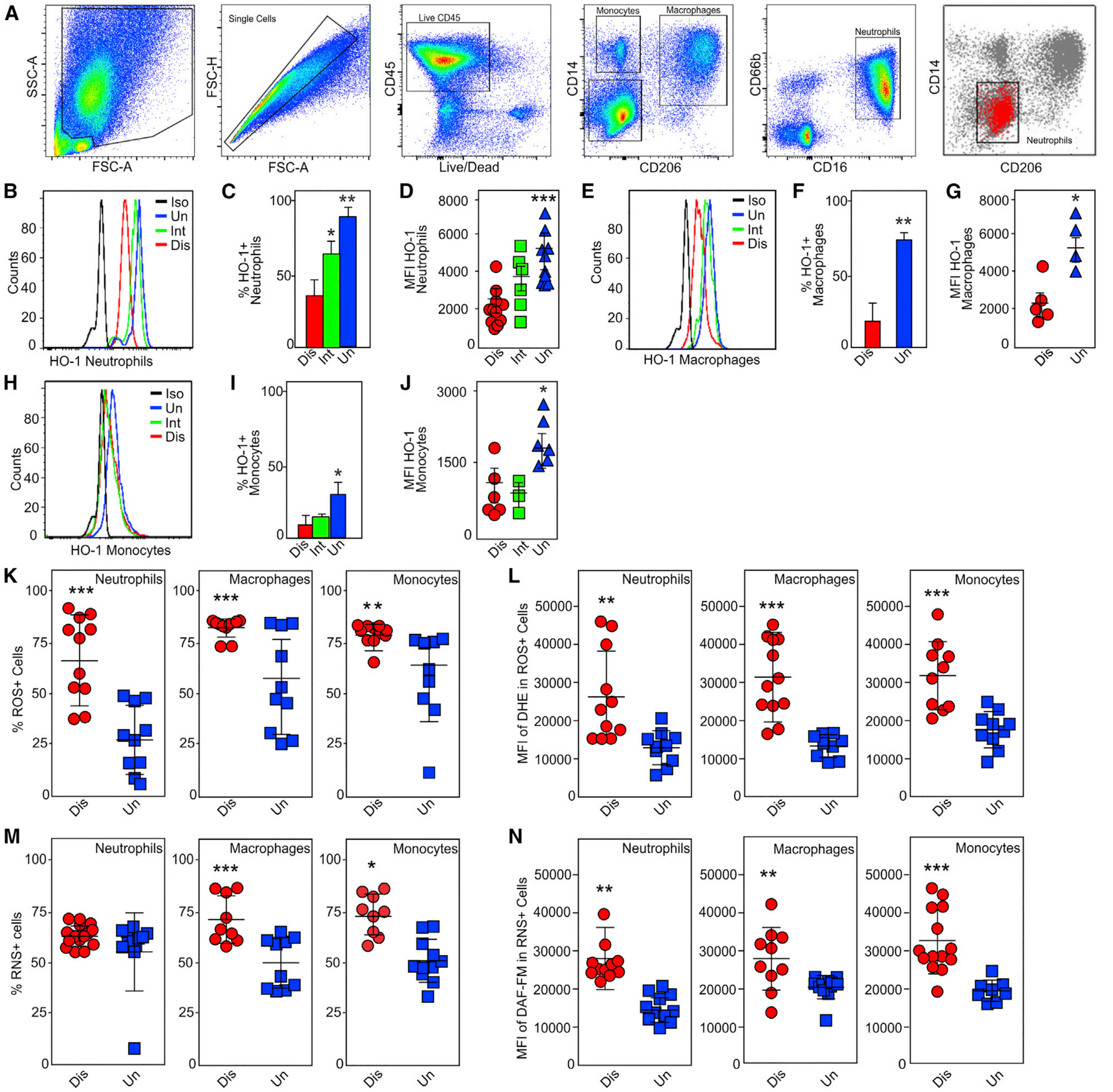


**Figure 3. HO-1, ROS, and RNS Levels in Myeloid Cells Isolated from Severely Diseased Regions of Human Tuberculous Lung Tissue**

(A) The sequential flow cytometry gating strategy for identification of monocytes, macrophages, neutrophils, and T cells from freshly isolated $CD45^{+}$ cells within the distinct pathological regions of human TB lung. Cells were identified by the following cell-surface markers: monocytes ($CD14^{+}HLA\text{-}DR^{+}CD11c^{low}CD66b^{-}CD3^{-}$), macrophages ($HLA\text{-}DR^{+}CD11c^{+}CD206^{+}CD11b^{+}CD86^{+}CD16^{-}CD66b^{-}CD3^{-}$), neutrophils ($CD11b^{+}CD66b^{+}CD16^{+}CD14^{-}CD3^{-}$), and T cells ($CD3^{+}CD14^{-}CD11b^{-}$).

(B) Representative histogram of HO-1 expression in neutrophils.

(C and D) Percentage of $HO\text{-}1^{+}$ neutrophils (C) and MFI of HO-1 in neutrophils (D).

(E) Representative histogram of HO-1 expression in macrophages.

(F and G) Percentage of $HO\text{-}1^{+}$ macrophages (F) and MFI of HO-1 in macrophages (G).

(H) Representative histogram of HO-1 expression in monocytes.

(I and J) Percentage of $HO\text{-}1^{+}$ monocytes (I) and MFI of HO-1 in monocytes (J).

(K and L) Percentage of ROS-positive neutrophils, macrophages, and monocytes (K) and their respective MFI of ROS (L).

(M and N) Percentage of RNS-positive neutrophils, macrophages, and monocytes (M) and their respective MFI within isolated lung immune cells (N).

n = 9–12 individual patients per group (each dot represents an individual patient). Statistical testing was performed using the unpaired Student's t test. Data are presented as mean ± SEM. $^{*}p < 0.05$, $^{**}p < 0.01$, $^{***}p < 0.001$. Black curve in (B), (E), and (H) represents isotype control (Iso).

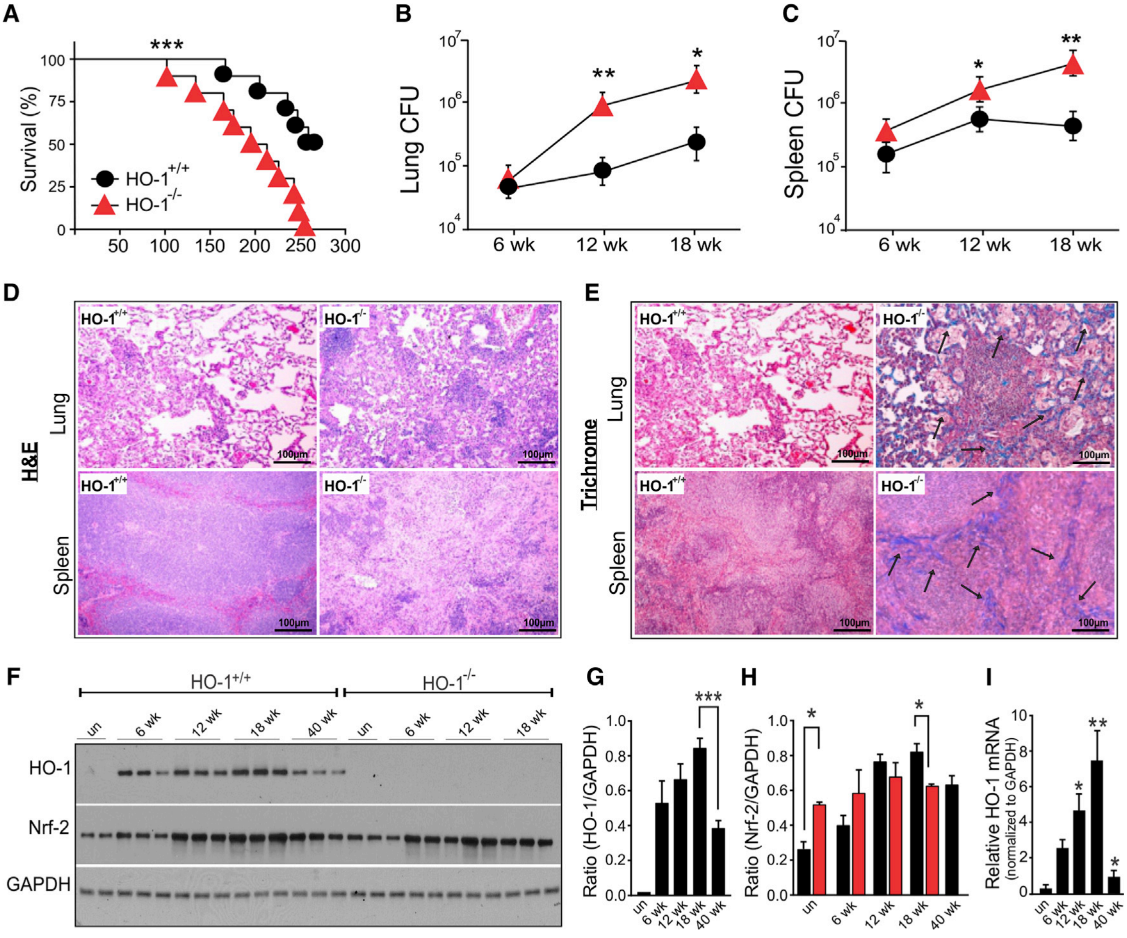


**Figure 4. HO-1$^{-/-}$ Mice Are More Susceptible to *Mtb* Infection**

(A) Kaplan-Meier survival analysis of HO-1$^{+/+}$ and HO-1$^{-/-}$ mice following infection with *Mtb* H37Rv. Uninfected HO-1$^{+/+}$ and HO-1$^{-/-}$ mice were used as controls and survived the duration of the study (data not shown). n = 10 mice per group.

(B and C) *Mtb* bacillary burden in lungs (B) and spleens (C) of HO-1$^{+/+}$ and HO-1$^{-/-}$ mice at 6, 12, and 18 weeks post-infection.

(D) H&E staining of representative lung and spleen sections from *Mtb*-infected HO-1$^{+/+}$ and HO-1$^{-/-}$ mice at 18 weeks post-infection.

(E) Trichrome staining of representative lung and spleen sections from *Mtb*-infected HO-1$^{+/+}$ and HO-1$^{-/-}$ mice at 18 weeks post-infection. Blue (as indicated by arrows) shows collagen deposition (scale bar, 100 μm).

(F) Western blot analysis of HO-1 and Nrf-2 in the total lung protein extract of uninfected and *Mtb*-infected HO-1$^{+/+}$ and HO-1$^{-/-}$ mice at 6, 12, 18, and 40 weeks post-infection. Each lane represents individual mice.

(G and H) Quantification of HO-1 (G) and Nrf-2 (H) western blots shown in (F) using ImageJ software. Target genes were normalized against the housekeeping gene GAPDH.

(I) Relative HO-1 mRNA expression in the lungs of *Mtb*-infected HO-1$^{+/+}$ mice was determined using qRT-PCR, normalized to the housekeeping gene β-actin. n = 4 for each time point. Statistical testing was performed using the unpaired Student's t test. Data are presented as mean ± SEM. *$p < 0.05$, **$p < 0.01$, ***$p < 0.001$.

isolated from severely diseased tissue compared to uninvolved lung (Figure 3K). We also observed a corresponding increase in the MFI of DHE fluorescence in these myeloid cell types (Figure 3L). Further, using the NO-reactive dye 4-amino-5-methylamino-2′,7′-difluorofluorescein (DAF-FM) diacetate, we detected significant increases in the percentages of reactive nitrogen species (RNS)-positive macrophages and monocytes isolated from severely diseased tissue compared to uninvolved tissue (Figure 3M). Interestingly, while there was no difference in the percentages of RNS-positive neutrophils based on tissue disease severity (Figure 3M), there was a significant increase in the corresponding MFI of DAF-FM in all three myeloid cell types isolated from diseased tissue (Figure 3N). Taken together, these data suggest that reduced levels of HO-1 and increased levels of ROS and RNS in myeloid cells are important contributors to TB immunopathology.

## HO-1$^{-/-}$ Mice Are More Susceptible to *Mtb* Infection

To investigate the role of HO-1 in TB disease progression and survival, we infected wild-type (HO-1$^{+/+}$) mice and mice completely deficient in HO-1 expression (HO-1$^{-/-}$). HO-1$^{-/-}$ mice were more susceptible to *Mtb* infection and began to succumb by 102 days post-infection, with a median survival of 204 days compared to 267 days for HO-1$^{+/+}$ mice (Figure 4A).

As expected, the bacillary load in the lungs (Figure 4B) and spleens (Figure 4C) was significantly higher in HO-1$^{-/-}$ mice than HO-1$^{+/+}$ mice at 12 and 18 weeks post-infection. The increased bacillary burden in HO-1$^{-/-}$ mice was accompanied by severe disease pathology (Figure 4D) and fibrosis (Figure 4E) at 18 weeks post-infection.

Our findings in human TB lungs showed that reduced HO-1 levels are associated with increased disease. Therefore, we tested whether HO-1 mRNA or protein levels change in the lung during *Mtb* infection in mice. In HO-1$^{+/+}$ mice, HO-1 mRNA (Figure 4I) and protein levels (Figures 4F and 4G) were increased at 6 weeks post-infection and continued to increase for at least 18 weeks. However, at a much later stage of the disease (40 weeks), HO-1 mRNA and protein levels were drastically reduced. To determine whether reduced levels of HO-1 at the late stage of infection were a consequence of changes in Nrf2, a master transcriptional regulator of oxidative-stress-related enzymes, we monitored Nrf2 levels over 40 weeks of infection. *Mtb* infection modestly increased Nrf2 protein levels in HO-1$^{+/+}$ mice at 18 weeks, which correlated with increases in HO-1 expression. However, at 40 weeks post-infection, Nrf2 levels remained elevated (Figures 4F and 4H) in contrast to HO-1 levels. Nrf2 levels were not markedly different between HO-1$^{-/-}$ and wild-type (WT) mice. Taken together, the data demonstrate that HO-1 expression is temporal and is necessary for host protection against *Mtb* disease. Also, reduced levels of HO-1 in mice correlate with increased disease severity. These findings are consistent with our human data and substantiate the importance of maintaining homeostatic levels of HO-1 in host protection against TB disease progression.

## Myeloid Cell Infiltration Is Dysregulated in *Mtb*-Infected HO-1$^{-/-}$ Mice

Since whole-blood transcriptome profiling has been proposed as a reliable measure of immune responses in TB patients (Berry et al., 2010), and because the plasma levels of HO-1 have been suggested as a potential TB diagnostic marker (Andrade et al., 2013; Andrade et al., 2014), we generated global transcriptomic profiles of blood monocytes from *Mtb*-infected mice. We identified 264 differentially regulated genes in *Mtb*-infected HO-1$^{-/-}$ mice (234 upregulated and 30 downregulated; Table S2). Using Ingenuity Pathway Analysis (IPA), we identified the top ten differentially regulated pathways in the categories of "immune cell trafficking" (Figure S7A) and "inflammatory responses" (Figure S7B) in HO-1$^{-/-}$ mice. The majority of these pathways control the movement, infiltration, and chemotaxis of different myeloid cell subsets, in particular neutrophils. Within these pathways, major pro-inflammatory genes such as *SOCS-3* and interleukin-12$\beta$ (*IL-12$\beta$*) were significantly upregulated whereas *IFN-$\gamma$* was significantly downregulated in *Mtb*-infected HO-1$^{-/-}$ mice (Figure S7C).

Next, to determine the differences in immune responses between *Mtb*-infected and uninfected HO-1$^{+/+}$ and HO-1$^{-/-}$ mice, we measured cytokine levels in the BALF. We observed no significant differences in cytokine levels between uninfected HO-1$^{+/+}$ and HO-1$^{-/-}$ mice. However, following infection, we observed significantly elevated levels of IL-1$\beta$, IL-2, IL-3, IL-5, IL-6, IL-10, IL-17A, MIP-1$\alpha$, MIP-1$\beta$, KC, transforming growth factor $\beta$ (TGF-$\beta$), G-CSF, and GM-CSF in HO-1$^{-/-}$ mice compared to HO-1$^{+/+}$ (Figures 5A–5M). Notably, these cytokines were also elevated in infected HO-1$^{LysM-/-}$ mice compared to the WT mice (Figures 7A–7K).

We next measured the accumulation of myeloid cells in infected HO-1$^{+/+}$ and HO-1$^{-/-}$ mice. We detected no difference in the percentage of macrophages (CD11b$^{+}$Ly6C) in the BALF or lungs from HO-1$^{-/-}$ and HO-1$^{+/+}$ mice (data not shown). However, we did detect significant increases in the percentage of neutrophils (CD11b$^{+}$Ly6G$^{+}$F4/80$^{-}$Gr-1$^{-}$) in the BALF and lungs of HO-1$^{-/-}$ mice compared to HO-1$^{+/+}$ mice at 6, 12, and 18 weeks post-infection (Figures 5N and 5O). We also saw increased percentages of monocytic (CD11b$^{+}$Ly6C$^{+}$F4/80$^{+}$ Gr-1$^{-}$) and granulocytic (CD11b$^{+}$Ly6G$^{+}$F/40$^{+}$Gr-1$^{+}$) myeloid cells in the lungs of *Mtb*-infected HO-1$^{-/-}$ mice. At 6 weeks post-infection, we detected no granulocytic myeloid cells; however, the percentage of monocytic cells in HO-1$^{-/-}$ mice was 5-fold higher than in HO-1$^{+/+}$ mice (Figures 5P and 5Q). Notably, at 12 and 18 weeks post-infection, the percentage of granulocytes was significantly higher in HO-1$^{-/-}$ mice than HO-1$^{+/+}$ mice and the monocyte population was no longer detectable, indicating a more granulocytic response with disease progression (Figures 5R and 5S). We also detected significantly increased accumulation of CD4$^{+}$FoxP3$^{+}$ T regulatory (T-reg) cells in the lungs of HO-1$^{-/-}$ mice (Figure S7D). Overall, these data are consistent with the known role of HO-1 in regulating myeloid cell infiltration (Hull et al., 2015; Tzima et al., 2009) and suggest that HO-1 controls myeloid cell infiltration and the associated inflammatory and cytokine responses to protect against *Mtb* disease progression.

## Mice Lacking HO-1 in Myeloid Cells Are More Susceptible to *Mtb* Infection

Our findings in human TB lung tissue (Figure 3) and full-body HO-1-deficient mice (Figure 4) suggest that homeostatic levels of HO-1 in myeloid cells are important for tolerance against TB. We then tested the hypothesis that HO-1 within myeloid cells protects against *Mtb* infection. We used mice expressing Cre recombinase (LysM-Cre) and biallelic floxed constructs (referred to as HO-1$^{LysM-/-}$) and control mice that lack LysM-Cre construct (referred to as HO-1$^{LysM+/+}$) (Hull et al., 2015). The survival of *Mtb*-infected HO-1$^{LysM-/-}$ mice was significantly reduced compared to HO-1$^{LysM+/+}$ mice (Figure 6A). Similarly, the bacterial burden in the lungs and spleens of HO-1$^{LysM-/-}$ mice was significantly greater at 12 and 22 weeks post-infection (Figures 6B and 6C). We measured immune cell infiltration and observed that while the absolute numbers of neutrophils and macrophages were not different, the percentages of neutrophils and macrophages were significantly higher in *Mtb*-infected HO-1$^{LysM-/-}$ mice than HO-1$^{LysM+/+}$ mice (Figures 6D–6H). This increase was also observed in uninfected mice, indicating that HO-1 expression in myeloid cells is important in myeloid cell migration, consistent with other reports (Hull et al., 2015; Tzima et al., 2009). We also observed significantly elevated levels of IL-1$\alpha$, IL-2, IL-3, IL-5, IL-6, IL-17A, MIP-1$\alpha$, MIP-1$\beta$, G-CSF, and KC in *Mtb*-infected HO-1$^{LysM-/-}$ mice compared to HO-1$^{LysM+/+}$ mice

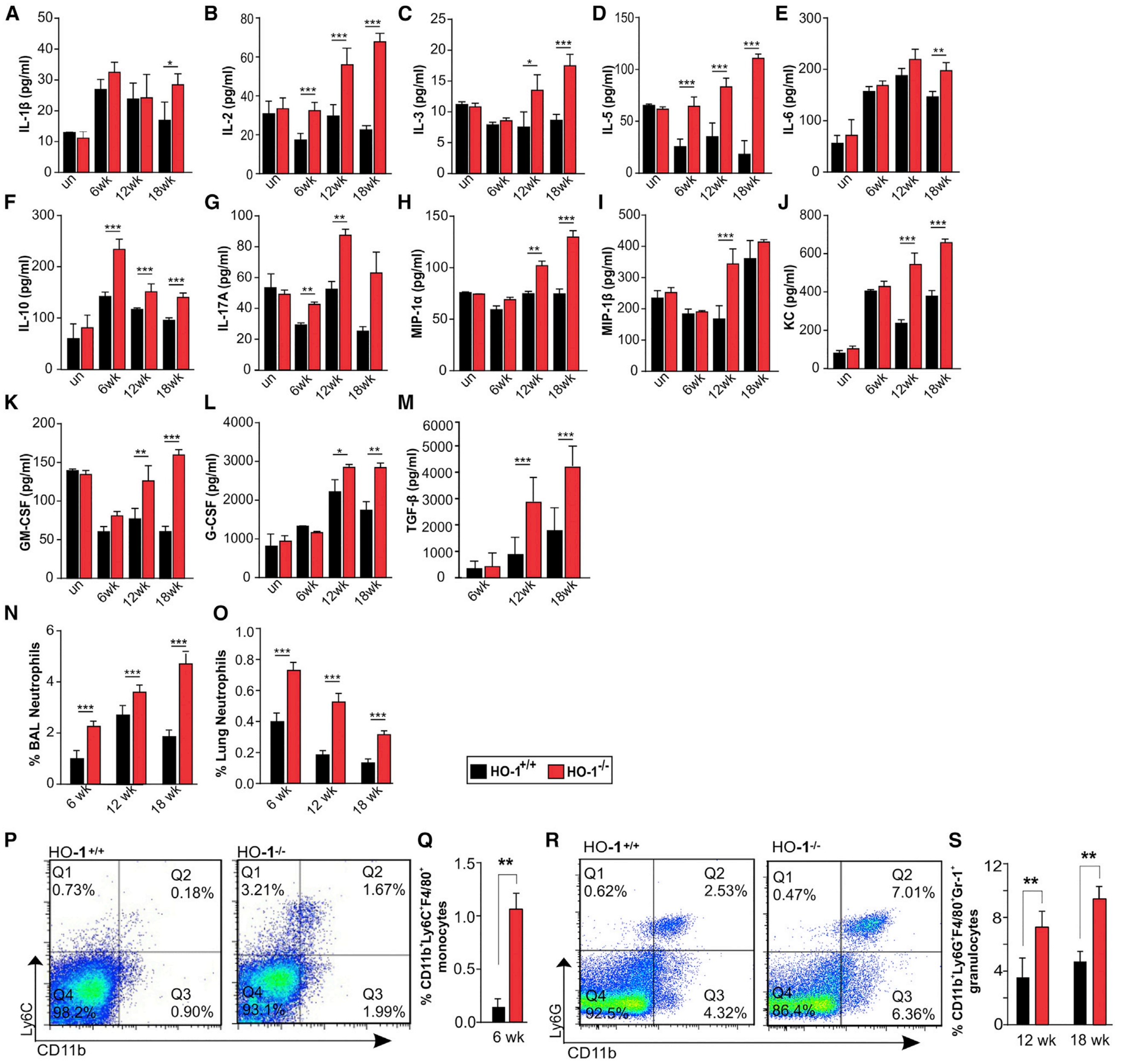


**Figure 5. Dysregulated Immune and Inflammatory Responses in *Mtb*-Infected HO-1$^{-/-}$ Mice**

(A–M) Cytokine analysis of IL-1β (A), IL-2 (B), IL-3 (C), IL-5 (D), IL-6 (E), IL-10 (F), IL-17A (G), MIP-1α (H), MIP-1β (I), KC (J), GM-CSF (K), G-CSF (L), TGF-β (M) in the BALF of uninfected and *Mtb*-infected HO-1$^{+/+}$ and HO-1$^{-/-}$ mice at 6, 12, and 18 weeks.

(N and O) Percentage of neutrophils in the BALF (N) and lungs (O) of *Mtb*-infected HO-1$^{+/+}$ and HO-1$^{-/-}$ mice at 6, 12, and 18 weeks post-infection.

(P–S) Other monocytes and granulocytes in the lungs of *Mtb*-infected HO-1$^{+/+}$ and HO-1$^{-/-}$ mice.

(P and Q) Representative scatterplot for CD11b$^{+}$Ly6C$^{+}$F4/80$^{+}$ monocytes (P) and percent differences (Q) at 6 weeks post-infection.

(R and S) Representative scatterplot for CD11b$^{+}$Ly6G$^{+}$F4/80$^{+}$Gr-1$^{+}$ granulocytes (R) and percent differences at 12 and 18 weeks post-infection (S).

n = 4 for each time point. Statistical testing was performed using the unpaired Student's t test. Data are presented as mean ± SEM. *$p < 0.05$, **$p < 0.01$, ***$p < 0.001$.

(Figures 7A–7K), consistent with our findings in HO-1$^{-/-}$ mice (Figures 5A–5M). Together, these results strongly support our hypothesis that HO-1 levels within myeloid cells play a key role in limiting lethal *Mtb* immunopathology and control overall *Mtb* disease progression.

## Myeloid HO-1 Deficiency Impairs IFN-γ Production by CD4$^{+}$ T Cells during *Mtb* Infection

To determine the effect of myeloid-specific HO-1 deficiency on adaptive immune responses, we measured responses in T cells isolated from the lungs of *Mtb*-infected HO-1$^{LysM-/-}$

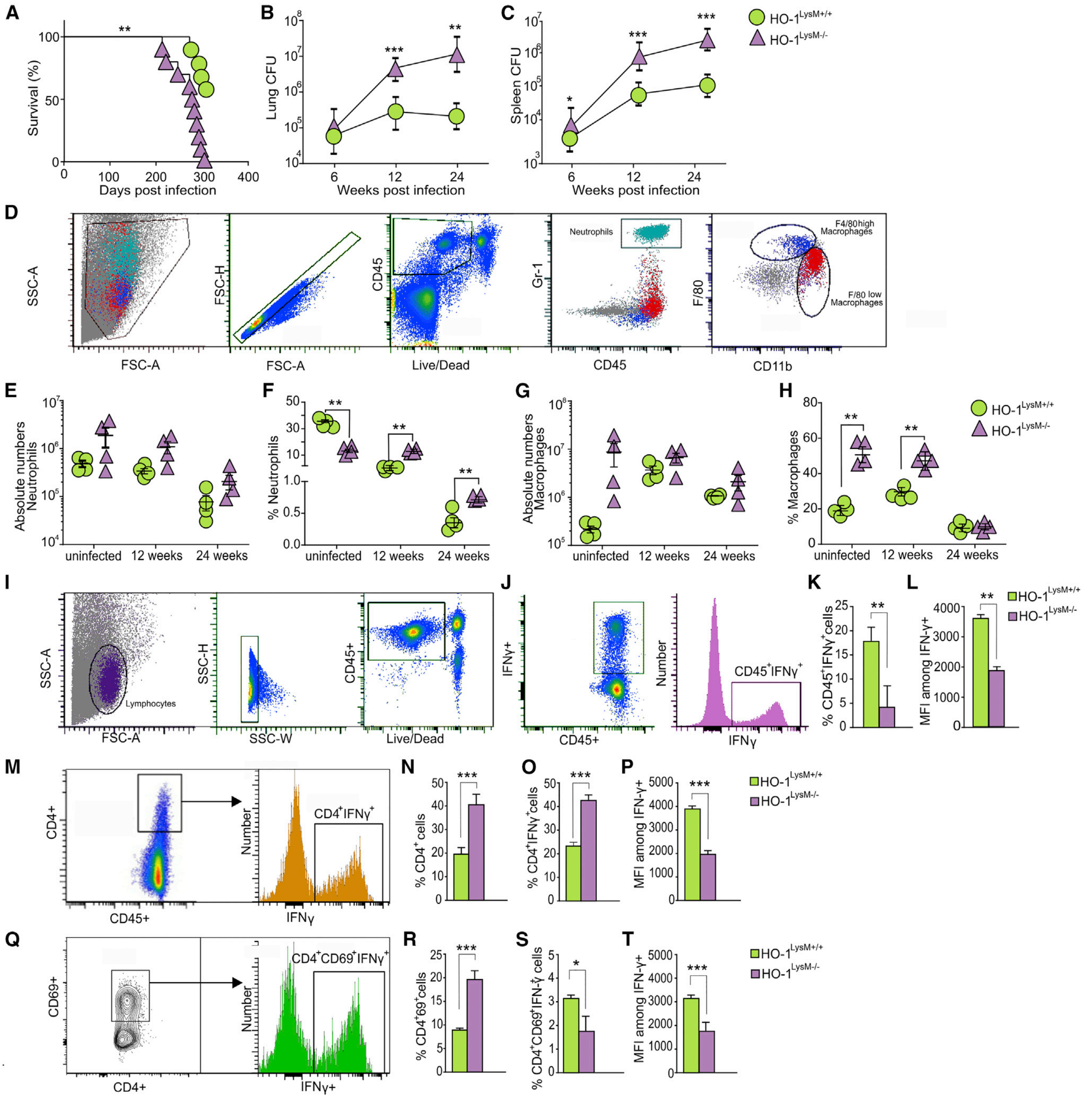


**Figure 6. Myeloid Specific HO-1 Knockout Mice (HO-1$^{LysM-/-}$) Are Susceptible to *Mtb* Infection**

(A) Kaplan-Meier survival analysis of *Mtb*-infected HO-1$^{LysM+/+}$ and HO-1$^{LysM}$ (n = 10 mice per group).

(B and C) Bacterial burden in lungs (B) and spleens (C) of *Mtb*-infected HO-1$^{LysM+/+}$ and HO-1$^{LysM-/-}$ mice at 6, 12, and 24 weeks post-infection.

(D) Flow cytometry gating strategy for identification of myeloid cells.

(E and F) Absolute numbers in cells per gram of tissue (E) and proportion of CD45$^{+}$ cells for lung neutrophils (F) from uninfected or *Mtb*-infected HO-1$^{LysM+/+}$ and HO-1$^{LysM-/-}$ mice at 12 and 24 weeks post-infection.

(G and H) Absolute numbers in cells per gram of tissue (G) and proportion of CD45$^{+}$ cells for lung macrophages (H) from uninfected or *Mtb*-infected HO-1$^{LysM+/+}$ and HO-1$^{LysM-/-}$ mice at 12 and 24 weeks post-infection.

(I) The sequential flow cytometry gating strategy for identification of IFN-γ-producing lymphocytes and T cells.

(J) Representative gated population of CD45$^{+}$IFN-γ$^{+}$ T cells.

(K and L) Differences in proportions of CD45$^{+}$ cells for IFN-γ$^{+}$ T cells (K) and IFN-γ MFI (L) isolated from the lungs of *Mtb*-infected HO-1$^{LysM+/+}$ and HO-1$^{LysM-/-}$ mice at 24 weeks post-infection.

(M) Representative gated population of CD45$^{+}$CD4$^{+}$IFN-γ$^{+}$ T cells.

*(legend continued on next page)*

mice (Figure 6I). We first measured IFN-$\gamma$ production in CD45$^+$ lymphocytes and observed that *Mtb*-infected HO-1$^{LysM-/-}$ mice have significantly reduced percentages of CD45$^+$IFN-$\gamma^+$ cells as well as reduced MFI of IFN-$\gamma$ (Figures 6J–6L). *Mtb*-infected HO-1$^{LysM-/-}$ mice also had increased percentages of CD4$^+$ T cells and significantly higher percentages of CD4$^+$IFN-$\gamma^+$-producing T cells (Figures 6M–6O). Surprisingly, the MFI of IFN-$\gamma$ in CD4$^+$ T cells was significantly lower in *Mtb*-infected HO-1$^{LysM-/-}$ mice than HO-1$^{LysM+/+}$ mice (Figure 6P). Further, *Mtb*-infected HO-1$^{LysM-/-}$ mice had increased percentages of activated (CD4$^+$CD69$^+$) T cells (Figures 6Q and 6R). We observed no significant differences in the percentages of IFN-$\gamma$-producing CD4$^+$CD69$^+$ T cells (Figure 6S). Interestingly, the MFI of IFN-$\gamma$ in activated T cells was significantly lower in *Mtb*-infected HO-1$^{LysM-/-}$ mice than HO-1$^{LysM+/+}$ mice (Figure 6T). Together, these results indicate that HO-1 in myeloid cells modulates IFN-$\gamma$ production by lymphocytes during *Mtb* infection.

## DISCUSSION

In this study, we demonstrate that HO-1 is essential for controlling myeloid cell inflammation and associated oxidative and/or nitrosative stress to protect against TB immunopathology in humans. We found that reduced levels of HO-1 result in elevated RNS and ROS levels in myeloid cells, increased neutrophil accumulation, karyorrhexis, and likely NETosis, thereby exacerbating tissue damage in human tuberculous lungs. In support of these findings, we employed HO-1-deficient mouse models and found that HO-1 reduces the levels of immunomodulatory cytokines and reduces the infiltration and accumulation of immune cells, including neutrophils, macrophages, and other myeloid cells. Further, the lack of HO-1 significantly reduced IFN-$\gamma$ production by T cells. Overall, this immune control by HO-1 results in decreased bacillary burden and increased survival in mice. Our findings point to distinct pathological and free-radical-mediated mechanisms whereby the temporal and spatial dysregulation of HO-1 within mouse and human lesions results in excessive inflammation and lethal immunopathology. The comprehensive characterization of immune cell types within the human TB lung represents an advance over TB studies that rely exclusively on human blood or BALF or on animal models that do not represent the full spectrum of human TB disease. Our findings establish a clinically relevant framework for understanding how *Mtb* causes active TB in humans and implicates HO-1 or its enzymatic products as potential therapeutic targets to impede TB disease progression (Figure 7L).

The role of HO-1 in TB has been the subject of much speculation. One study has shown that HO-1 is protective during mycobacterial infections, including *Mtb* (Silva-Gomes et al., 2013). However, a small number of mice were used in these survival studies, and it could be argued that abnormalities associated with whole-body HO-1-deficient mice confounded the *Mtb* infection studies. Our survival studies used a suitable number (n = 12) of HO-1$^{-/-}$ mice and provide strong evidence that HO-1 is essential for protection against *Mtb* infection. In addition, our myeloid-specific knockout mice, which exhibit no signs of abnormality (Tzima et al., 2009), were more susceptible to *Mtb* infection with significantly increased bacterial burden and reduced survival. In contrast, it was reported that pharmacological inhibition of HO-1 (Costa et al., 2016; Scharn et al., 2016) results in reduced *Mtb* burden, suggesting that HO-1 promotes TB. However, we view these findings as inconclusive, since pharmacological inhibition of HO-1 had no effect on the survival of *Mtb*-infected mice (Costa et al., 2016), suggesting that other factors such as HO-1-independent T cell receptor mechanisms may be involved. Further, the suggestion that HO-1 inhibition and a subsequent increase in the localized heme concentration could aid in oxidative-stress-mediated killing of *Mtb* overlooks the widely known fact the heme itself can cause massive tissue damage. Indeed, pulmonary hemorrhage (Reddy et al., 2018) and excessive hemoptysis, which release heme, are key features of acute human TB. In addition, the importance of HO-1 in human health is accentuated by HO-1 deficiency studies showing systemic inflammation, red blood cell fragility, intravascular hemolysis, hemorrhage, endothelial damage, asplenia, remarkable vulnerability to infection, high concentrations of extracellular heme, and early death (Kawashima et al., 2002; Radhakrishnan et al., 2011). Lastly, HO-1 deficiency in humans causes a more severe phenotype than in mice (Kawashima et al., 2002; Yachie et al., 1999), which is supported by the fact that unlike humans (Radhakrishnan et al., 2011), mice fully deficient in HO-1 have a normal lifespan, albeit with some abnormalities (Fraser et al., 2011).

Why is it necessary to examine HO-1 within the pathological spectrum of human TB? First, it is reasonable to expect that the spectrum correlates with the clinical, immunological, and pathological diversity of TB (Brostoff et al., 1981; Lenzini et al., 1977). Therefore, characterizing HO-1 within the diversity of microanatomical niches generated by the bacillus, host, or both, versus a single snapshot within the spectrum, will more accurately contextualize HO-1-mediated cytoprotection during TB. Second, as highlighted elsewhere (Cadena et al., 2017; Kaplan et al., 2003; Kim et al., 2010), immunological variability that contributes to a range of lesions presents the bacilli with a choice of fuel sources that almost certainly contributes to the dynamics of disease progression. Lastly, relating the microanatomic architecture to the underlying immune state of the TB patient may suggest alternative therapeutic strategies and

(N) Differences in proportions of CD45$^+$ cells for CD4$^+$ T cells in the lungs of *Mtb*-infected HO-1$^{LysM+/+}$ and HO-1$^{LysM-/-}$ mice at 24 weeks post-infection.

(O and P) Differences in proportions of CD45$^+$ cells (O) and IFN-$\gamma$ MFI (P) for CD45$^+$CD4$^+$IFN-$\gamma^+$ T cells isolated from the lungs of *Mtb*-infected HO-1$^{LysM+/+}$ and HO-1$^{LysM-/-}$ mice at 24 weeks post-infection.

(Q) Representative gated population of CD45$^+$CD4$^+$CD69$^+$IFN-$\gamma^+$ T cells.

(R) Differences in proportions of CD45$^+$ cells for CD45$^+$CD4$^+$CD69$^+$ T cells in the lungs of *Mtb*-infected HO-1$^{LysM+/+}$ and HO-1$^{LysM-/-}$ mice at 24 weeks post-infection.

(S and T) Differences in proportions of CD45$^+$ cells (S) and IFN-$\gamma$ MFI (T) for CD45$^+$CD4$^+$CD69$^+$IFN-$\gamma^+$ T cells isolated from the lungs of *Mtb*-infected HO-1$^{LysM+/+}$ and HO-1$^{LysM-/-}$ mice at 24 weeks post-infection.

n = 4 for each time point. Statistical testing was performed using the unpaired Student's t test. Data are presented as mean ± SEM. *p < 0.05, **p < 0.01, ***p < 0.001.

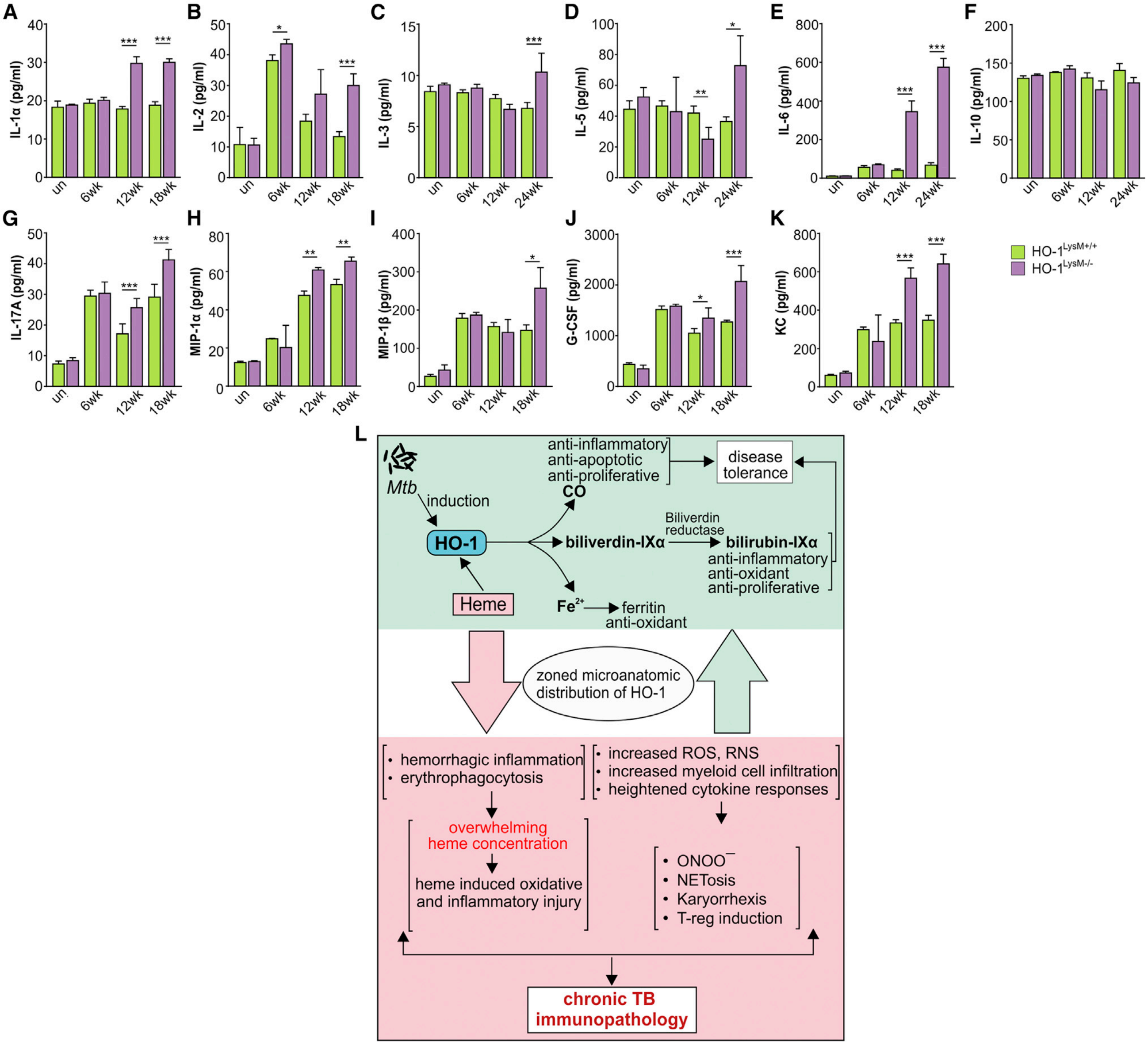


**Figure 7. Cytokine Response in HO-1 LysM Mice and Proposed Model for HO-1-Mediated Protection against *Mtb* Infection**

(A–K) Cytokine analysis (A), IL-2 (B), IL-3 (C), IL-5 (D), IL-6 (E), IL-10 (F), IL-17A (G), MIP-1α (H), MIP-1β (I), G-CSF (J), KC (K) in the BALF of uninfected and *Mtb*-infected HO-1$^{LysM+/+}$ and HO-1$^{LysM-/-}$ mice at 6, 12, and 18 weeks post-infection. n = 4 for each time point. Statistical testing was performed using the unpaired Student's t test. Data are presented as mean ± SEM. *p < 0.05, **p < 0.01, ***p < 0.001.

(L) Proposed model for HO-1-mediated protection during *Mtb* infection. *Mtb* infection induces HO-1 expression and its downstream enzymatic cascade. Together with its enzymatic products and ferritin, which is induced by HO-1, HO-1 protects against TB immunopathology and thereby contributes to overall TB disease tolerance via its anti-oxidant, anti-inflammatory, and anti-proliferative properties. Therefore, maintaining the physiological levels of HO-1 is important to limit TB disease pathology (green shaded area). Contrarily, in HO-1-deficient conditions, the levels of key pro-inflammatory cytokines are significantly elevated. This results in rapid and dysregulated myeloid cell infiltration to the infected sites, which results in significantly elevated levels of ROS and RNS as well as rapid karyorrhexis and NETosis. ROS and RNS also generate increased localized concentrations of peroxynitrite ($ONOO^-$) and subsequent suppression of T cell responses. In addition, *Mtb* infection also causes endothelial injury and intravascular coagulation, resulting in significant accumulation of heme, a potent proinflammatory and pro-oxidant molecule. Excess heme overwhelms the cytoprotective activity of HO-1, thereby contributing pro-oxidant-mediated inflammatory responses. Together, these dysregulated inflammatory responses result in chronic TB immunopathology and rapid disease progression (red shaded area).

will provide important benchmarks for validation of animal models of disease.

A common feature that emerged from our studies is the spatiotemporal production of HO-1. In WT infected mice, an initial increase in HO-1 levels was followed by a progressive decrease that correlates with increased mortality, pointing to the protective role of HO-1. Similarly, our IHC and flow cytometry data from human tuberculous lungs show that cellular distribution

and cell type (e.g. neutrophils) and levels of HO-1 in and around human TB lesions regulate disease progression. HO-1 is expressed within healthy cellular compartments in normal (Figure S4F) and TB-afflicted lungs (Figures 1 and 2), but the cellular distribution and levels differ. We found that neutrophils and macrophages are the predominant HO-1-producing immune cell type in human TB lungs. Of note were the significantly reduced levels of neutrophil HO-1 in the most diseased tissue, signifying a loss of cytoprotection. These observations clearly implicate HO-1 as a therapeutic target to improve TB disease pathology. Although increases in myeloid cell infiltration have been reported in mouse models of TB and in TB patients (du Plessis et al., 2013; Gopal et al., 2013), the host factors regulating their migration and accumulation are poorly defined. Further, the role of myeloid cells during active TB remains unclear. Our IPA pathway analysis of *Mtb*-infected HO-$1^{-/-}$ and HO-$1^{+/+}$ mice demonstrate that HO-1 is a key regulatory factor in myeloid cell migration, especially neutrophils, which is consistent with its role in regulating migration of myeloid cells (Freitas et al., 2006; Hull et al., 2015). We observed significantly elevated levels of pro-inflammatory cytokines, including IL-6, KC, TGF-β, IL-1α, and granulocyte/monocyte colony-stimulating factors such as G-CSF and GM-CSF, in the BALF of HO-$1^{-/-}$ mice compared to HO-$1^{+/+}$ mice. These cytokines modulate chemotaxis, induction, and migration of distinct myeloid cells in many pathological conditions (Hammond et al., 1995; McLoughlin et al., 2003), consistent with our IPA data (Figures S7A–S7C). We consistently observed increased accumulation of these myeloid cells in the lungs of both HO-1-deficient mouse models. We also observed neutrophil accumulation in human TB lungs (Figure S6A) as well as the co-localization of NE, MPO, and histone H2A, suggesting the formation of NETs (Papayannopoulos et al., 2010). This supports other *in vitro* studies demonstrating that *Mtb* induces NET formation and suggests a role for NETs in human TB (Braian et al., 2013).

What are the factors that influence HO-1 cytoprotection during TB? First, we found that myeloid cells in severely diseased TB lung tissue produce large quantities of ROS and RNS and significantly reduced HO-1 levels. We also observed increased Arg-1 levels in human TB lung and in *Mtb*-infected $HO^{-/-}$ mice. Activated macrophages induce iNOS and resultant RNS and/or NO, whereas neutrophils induce NADPH oxidase to generate ROS and/or $O_2^{\bullet-}$. These factors are major contributors to immunosuppression via induction of T-regs and inhibition of cytotoxic T lymphocytes, dendritic cells, and natural killer cells (Gabrilovich and Nagaraj, 2009; Pillay et al., 2013). Neutrophils produce superoxide dismutase that converts $O_2^{\bullet-}$ into $H_2O_2$, which is a potent suppressor of T cell functions (Bowers et al., 2014). However, the decreased Th1 response and increased T-reg accumulation observed in HO-1-deficient mice point to a mechanism wherein the reduced HO-1 in myeloid cells triggers immune suppression and tissue damage. As expected, the percentage of granulocytes in HO-$1^{-/-}$ mice increased during disease progression (Figures 5N–5S). Neutrophils generate high levels of $O_2^{\bullet-}$ and low levels of NO, whereas macrophages generate low levels of $O_2^{\bullet-}$ and high NO levels. NO reacts with $O_2^{\bullet-}$ to generate $ONOO^-$, a potent suppressor of T cell function and bactericidal free radical (Nagaraj and Gabrilovich, 2008). Hence, the ratio of granulocytes to monocytes producing $O_2^{\bullet-}$ and NO, respectively, to generate $ONOO^-$ has important pathological consequences during TB disease progression. Second, it is well established that oxidative stress can trigger karyorrhexis (Korolczuk et al., 2016), a process that overlaps with apoptosis and NETosis that we observed in the human tuberculous lung (Figures S6A and S6B). Not surprisingly, large numbers of karyorrhectic, degenerate neutrophils were observed in cavitary lesions and around caseative suppurative granulomatous lesions, which upon HO-1 staining produced a clearly defined zonal or graded HO-1 pattern (Figures 1, 2, S2A, S4D, and S4E). Third, consistent with previous studies and our histopathological analyses, we observed substantial hemorrhagic inflammation (Figures S6C, S1A, and S1B), resulting from ruptured alveolar walls and the release of large quantities of erythrocyte-derived heme (Canetti, 1955). As previously reported, excess heme and release of iron (Reddy et al., 2018) may overwhelm the cytoprotective capacity of HO-1 by rapid recruitment of neutrophils, oxidative and inflammatory imbalance, and vascular dysfunction (Jeney et al., 2002; Wagener et al., 2001). This pathophysiological response may be exacerbated by the decreased HO-1 levels we observed in human TB lung tissue and in animal studies at the late stages of infection. Therefore, it is not surprising that fluctuations in HO-1 levels in TB foci lead to dysregulated immune responses. Overall, our findings suggest that reduced HO-1 levels lead to increased migration and accumulation of inflammatory myeloid cells, including neutrophils, which leads to increased ROS, RNS, and Arg-1 levels. In turn, this uncontrolled inflammation results in immunosuppression and disease pathology.

A potential limitation of our human studies is that lung tissue was sourced from TB patients with different medical histories and treatments. Indeed, this may be reflected in the inconsistent macrophage population observed in some of the human lung tissues. However, the statistically significant differences in HO-1 levels, ROS, and RNS in neutrophils, macrophages, and monocytes isolated from diseased, intermediate, and uninvolved lung tissues, albeit from different patients, highlight the critical role of HO-1 in mediating the inflammatory response in human TB. Lastly, it is likely that a larger test cohort may render a wider and more divergent disease spectrum, which is the goal of ongoing studies.

In conclusion, our data demonstrate that HO-1 expression in myeloid cells is crucial for host defense to limit immunopathology and disease progression during *Mtb* infection. The evidence shows that zonal HO-1 distribution within the TB microenvironment regulates the accumulation and inflammatory responses of myeloid cells. These responses include RNS- and ROS-mediated stress, karyorrhexis, NETosis, and intravascular hemorrhage, which overall dictate the TB immunopathology. Lastly, these findings suggest that pharmacological upregulation of cytoprotective HO-1 activity may limit immunopathology during active TB disease.

## STAR★METHODS

Detailed methods are provided in the online version of this paper and include the following:

- KEY RESOURCES TABLE
- LEAD CONTACT FOR REAGENT AND RESOURCE SHARING

- EXPERIMENTAL MODEL AND SUBJECT DETAILS
  - Human Subjects
  - Animals
  - Bacterial Strains
- METHOD DETAILS
  - Infection of Mice
  - Microarray and Pathway Analysis
  - Determination of Bacterial Burden
  - Isolation of BAL fluid and Cytokine ELISA
  - Western Blotting
  - RNA extraction and real-time PCR from lung tissues
  - Mouse Histopathology
  - Flow Cytometry of Mice Immune Cell Isolates
  - Human Lung Cell Isolation and Flow Cytometry
  - Human Lung Tissue Immunohistochemistry
  - Spatial image analysis of HO-1 distribution within human TB granulomas
- QUANTIFICATION AND STATISTICAL ANALYSIS
- DATA AND SOFTWARE AVAILABILITY

## SUPPLEMENTAL INFORMATION

Supplemental Information includes seven figures and two tables and can be found with this article online at https://doi.org/10.1016/j.celrep.2018.10.073.

## ACKNOWLEDGMENTS

This work was supported by NIH grants R01AI111940 and R21A127182 and Bill and Melinda Gates Foundation award OPP1130017 (to A.J.C.S.). This work was also supported by NIH grant DK59600 (to A.A.), the UAB Center for AIDS Research (CFAR), and the Center for Free Radical Biology (CFRB) (A.J.C.S.). The research was also co-funded by the South African Medical Research Council (A.J.C.S.).

## AUTHOR CONTRIBUTIONS

Conceptualization and Methodology, K.C.C. and A.J.C.S.; Resources – Human TB Lung, R.M.W. and R.M.; Resources – Animals, A.A. and A.T.; Immunohistochemistry – Humans, P.K.R., G.W., and K.N.; Immunohistochemistry – Animals, K.C.C. and G.P.S.; Animal Studies, K.C.C., V.S., V.P.R., and J.N.G.; Flow Cytometry – Animals, K.C.C., J.M.L., J.D., and J.F.G.; Flow Cytometry – Humans, M.A.R., S.N., and A.L.; Microarray, V.S. and V.B.A.; Writing – Original Draft, K.C.C. and A.J.C.S.; Writing – Review & Editing, K.C.C., J.N.G., and A.J.C.S.; Funding, A.J.C.S and A.A. All authors discussed the results and commented on the manuscript.

## DECLARATION OF INTERESTS

The authors declare no competing interests.

Received: October 30, 2017
Revised: July 18, 2018
Accepted: October 19, 2018
Published: November 13, 2018

## REFERENCES

Andrade, B.B., Pavan Kumar, N., Mayer-Barber, K.D., Barber, D.L., Sridhar, R., Rekha, V.V.B., Jawahar, M.S., Nutman, T.B., Sher, A., and Babu, S. (2013). Plasma heme oxygenase-1 levels distinguish latent or successfully treated human tuberculosis from active disease. PLoS ONE *8*, e62618.

Andrade, B.B., Kumar, N.P., Sridhar, R., Banurekha, V.V., Jawahar, M.S., Nutman, T.B., Sher, A., and Babu, S. (2014). Heightened plasma levels of heme oxygenase-1 and tissue inhibitor of metalloproteinase-4 as well as elevated peripheral neutrophil counts are associated with TB-diabetes comorbidity. Chest *145*, 1244–1254.

Andrade, B.B., Pavan Kumar, N., Amaral, E.P., Riteau, N., Mayer-Barber, K.D., Tosh, K.W., Maier, N., Conceição, E.L., Kubler, A., Sridhar, R., et al. (2015). Heme oxygenase-1 regulation of matrix metalloproteinase-1 expression underlies distinct disease profiles in tuberculosis. J. Immunol. *195*, 2763–2773.

Barksdale, S.K., Scumpia, P., Wang, J.Y., Xu, X., and Barnhill, R.L. (2015). Vascular disease. In Lever's Histopathology of the Skin, Eleventh Edition, D.E. Elder, R. Elinitsas, M. Rosenbach, G.F. Murphy, A.I. Rubin, and X. Xu, eds. (Lippincott William & Wilkins), pp. 240–275.

Barry, C.E., 3rd, Boshoff, H.I., Dartois, V., Dick, T., Ehrt, S., Flynn, J., Schnappinger, D., Wilkinson, R.J., and Young, D. (2009). The spectrum of latent tuberculosis: rethinking the biology and intervention strategies. Nat. Rev. Microbiol. *7*, 845–855.

Berry, M.P., Graham, C.M., McNab, F.W., Xu, Z., Bloch, S.A., Oni, T., Wilkinson, K.A., Banchereau, R., Skinner, J., Wilkinson, R.J., et al. (2010). An interferon-inducible neutrophil-driven blood transcriptional signature in human tuberculosis. Nature *466*, 973–977.

Bolisetty, S., Zarjou, A., Hull, T.D., Traylor, A.M., Perianayagam, A., Joseph, R., Kamal, A.I., Arosio, P., Soares, M.P., Jeney, V., et al. (2015). Macrophage and epithelial cell H-ferritin expression regulates renal inflammation. Kidney Int. *88*, 95–108.

Bowers, N.L., Helton, E.S., Huijbregts, R.P.H., Goepfert, P.A., Heath, S.L., and Hel, Z. (2014). Immune suppression by neutrophils in HIV-1 infection: role of PD-L1/PD-1 pathway. PLoS Pathog. *10*, e1003993.

Braian, C., Hogea, V., and Stendahl, O. (2013). *Mycobacterium tuberculosis*-induced neutrophil extracellular traps activate human macrophages. J. Innate Immun. *5*, 591–602.

Brostoff, J., Lenzini, L., Rottoli, P., and Rottoli, L. (1981). Immune complexes in the spectrum of tuberculosis. Tubercle *62*, 169–173.

Cadena, A.M., Fortune, S.M., and Flynn, J.L. (2017). Heterogeneity in tuberculosis. Nat. Rev. Immunol. *17*, 691–702.

Canetti, G. (1955). The Tubercle Bacillus in the Pulmonary Lesion of Man: Histobacteriology and Its Bearing on the Therapy of Pulmonary Tuberculosis (Springer Publishing Company).

Castilho, Á., Aveleira, C.A., Leal, E.C., Simões, N.F., Fernandes, C.R., Meirinhos, R.I., Baptista, F.I., and Ambrósio, A.F. (2012). Heme oxygenase-1 protects retinal endothelial cells against high glucose- and oxidative/nitrosative stress-induced toxicity. PLoS ONE *7*, e42428.

Chinta, K.C., Saini, V., Glasgow, J.N., Mazorodze, J.H., Rahman, M.A., Reddy, D., Lancaster, J.R., Jr., and Steyn, A.J. (2016). The emerging role of gasotransmitters in the pathogenesis of tuberculosis. Nitric Oxide *59*, 28–41.

Choi, A.M., and Alam, J. (1996). Heme oxygenase-1: function, regulation, and implication of a novel stress-inducible protein in oxidant-induced lung injury. Am. J. Respir. Cell Mol. Biol. *15*, 9–19.

Costa, D.L., Namasivayam, S., Amaral, E.P., Arora, K., Chao, A., Mittereder, L.R., Maiga, M., Boshoff, H.I., Barry, C.E., 3rd, Goulding, C.W., et al. (2016). Pharmacological inhibition of host heme oxygenase-1 suppresses *Mycobacterium tuberculosis* infection *in vivo* by a mechanism dependent on T lymphocytes. MBio *7*, e01675–e16.

Datta, P.K., Koukouritaki, S.B., Hopp, K.A., and Lianos, E.A. (1999). Heme oxygenase-1 induction attenuates inducible nitric oxide synthase expression and proteinuria in glomerulonephritis. J. Am. Soc. Nephrol. *10*, 2540–2550.

Dharmadhikari, A.S., and Nardell, E.A. (2008). What animal models teach humans about tuberculosis. Am. J. Respir. Cell Mol. Biol. *39*, 503–508.

du Plessis, N., Loebenberg, L., Kriel, M., von Groote-Bidlingmaier, F., Ribechini, E., Loxton, A.G., van Helden, P.D., Lutz, M.B., and Walzl, G. (2013). Increased frequency of myeloid-derived suppressor cells during active tuberculosis and after recent *mycobacterium tuberculosis* infection suppresses T-cell function. Am. J. Respir. Crit. Care Med. *188*, 724–732.

Duque-Correa, M.A., Kühl, A.A., Rodriguez, P.C., Zedler, U., Schommer-Leitner, S., Rao, M., Weiner, J., 3rd, Hurwitz, R., Qualls, J.E., Kosmiadi, G.A., et al. (2014).

Macrophage arginase-1 controls bacterial growth and pathology in hypoxic tuberculosis granulomas. Proc. Natl. Acad. Sci. USA *111*, E4024–E4032.

Fraser, S.T., Midwinter, R.G., Berger, B.S., and Stocker, R. (2011). Heme oxygenase-1: A critical link between iron metabolism, erythropoiesis, and development. Adv. Hematol. *2011*, 473709.

Freitas, A., Alves-Filho, J.C., Secco, D.D., Neto, A.F., Ferreira, S.H., Barja-Fidalgo, C., and Cunha, F.Q. (2006). Heme oxygenase/carbon monoxide-biliverdin pathway down regulates neutrophil rolling, adhesion and migration in acute inflammation. Br. J. Pharmacol. *149*, 345–354.

Gabrilovich, D.I., and Nagaraj, S. (2009). Myeloid-derived suppressor cells as regulators of the immune system. Nat. Rev. Immunol. *9*, 162–174.

George, J.F., Braun, A., Brusko, T.M., Joseph, R., Bolisetty, S., Wasserfall, C.H., Atkinson, M.A., Agarwal, A., and Kapturczak, M.H. (2008). Suppression by CD4+CD25+ regulatory T cells is dependent on expression of heme oxygenase-1 in antigen-presenting cells. Am. J. Pathol. *173*, 154–160.

Gopal, R., Monin, L., Torres, D., Slight, S., Mehra, S., McKenna, K.C., Fallert Junecko, B.A., Reinhart, T.A., Kolls, J., Báez-Saldaña, R., et al. (2013). S100A8/A9 proteins mediate neutrophilic inflammation and lung pathology during tuberculosis. Am. J. Respir. Crit. Care Med. *188*, 1137–1146.

Hammond, M.E., Lapointe, G.R., Feucht, P.H., Hilt, S., Gallegos, C.A., Gordon, C.A., Giedlin, M.A., Mullenbach, G., and Tekamp-Olson, P. (1995). IL-8 induces neutrophil chemotaxis predominantly via type I IL-8 receptors. J. Immunol. *155*, 1428–1433.

Hull, T.D., Kamal, A.I., Boddu, R., Bolisetty, S., Guo, L., Tisher, C.C., Rangarajan, S., Chen, B., Curtis, L.M., George, J.F., and Agarwal, A. (2015). Heme oxygenase-1 regulates myeloid cell trafficking in AKI. J. Am. Soc. Nephrol. *26*, 2139–2151.

Jais, A., Einwallner, E., Sharif, O., Gossens, K., Lu, T.T., Soyal, S.M., Medgyesi, D., Neureiter, D., Paier-Pourani, J., Dalgaard, K., et al. (2014). Heme oxygenase-1 drives metaflammation and insulin resistance in mouse and man. Cell *158*, 25–40.

Jeney, V., Balla, J., Yachie, A., Varga, Z., Vercellotti, G.M., Eaton, J.W., and Balla, G. (2002). Pro-oxidant and cytotoxic effects of circulating heme. Blood *100*, 879–887.

Kaplan, G., Post, F.A., Moreira, A.L., Wainwright, H., Kreiswirth, B.N., Tanverdi, M., Mathema, B., Ramaswamy, S.V., Walther, G., Steyn, L.M., et al. (2003). *Mycobacterium tuberculosis* growth at the cavity surface: a microenvironment with failed immunity. Infect. Immun. *71*, 7099–7108.

Kawashima, A., Oda, Y., Yachie, A., Koizumi, S., and Nakanishi, I. (2002). Heme oxygenase-1 deficiency: the first autopsy case. Hum. Pathol. *33*, 125–130.

Kim, M.-J., Wainwright, H.C., Locketz, M., Bekker, L.-G., Walther, G.B., Dittrich, C., Visser, A., Wang, W., Hsu, F.-F., Wiehart, U., et al. (2010). Caseation of human tuberculosis granulomas correlates with elevated host lipid metabolism. EMBO Mol. Med. *2*, 258–274.

Korolczuk, A., Caban, K., Amarowicz, M., Czechowska, G., and Irla-Miduch, J. (2016). Oxidative stress and liver morphology in experimental cyclosporine A-induced hepatotoxicity. BioMed Res. Int. *2016*, 5823271.

Kumar, A., Deshane, J.S., Crossman, D.K., Bolisetty, S., Yan, B.S., Kramnik, I., Agarwal, A., and Steyn, A.J. (2008). Heme oxygenase-1-derived carbon monoxide induces the *Mycobacterium tuberculosis* dormancy regulon. J. Biol. Chem. *283*, 18032–18039.

Laga, A.C., and Milner, D.A., Jr. (2015). Bacterial diseases. In Lever's Histopathology of the Skin, 11th edition, D.E. Elder, R. Elinitsas, M. Rosenbach, G.F. Murphy, A.I. Rubin, and X. Xu, eds. (Lippincott William & Wilkins), pp. 658–713.

Lenzini, L., Rottoli, P., and Rottoli, L. (1977). The spectrum of human tuberculosis. Clin. Exp. Immunol. *27*, 230–237.

McLoughlin, R.M., Witowski, J., Robson, R.L., Wilkinson, T.S., Hurst, S.M., Williams, A.S., Williams, J.D., Rose-John, S., Jones, S.A., and Topley, N. (2003). Interplay between IFN-gamma and IL-6 signaling governs neutrophil trafficking and apoptosis during acute inflammation. J. Clin. Invest. *112*, 598–607.

Nagaraj, S., and Gabrilovich, D.I. (2008). Tumor escape mechanism governed by myeloid-derived suppressor cells. Cancer Res. *68*, 2561–2563.

Obregón-Henao, A., Henao-Tamayo, M., Orme, I.M., and Ordway, D.J. (2013). Gr1(int)CD11b+ myeloid-derived suppressor cells in Mycobacterium tuberculosis infection. PLoS ONE *8*, e80669.

Papayannopoulos, V., Metzler, K.D., Hakkim, A., and Zychlinsky, A. (2010). Neutrophil elastase and myeloperoxidase regulate the formation of neutrophil extracellular traps. J. Cell Biol. *191*, 677–691.

Pillay, J., Tak, T., Kamp, V.M., and Koenderman, L. (2013). Immune suppression by neutrophils and granulocytic myeloid-derived suppressor cells: similarities and differences. Cell. Mol. Life Sci. *70*, 3813–3827.

Poss, K.D., and Tonegawa, S. (1997). Heme oxygenase 1 is required for mammalian iron reutilization. Proc. Natl. Acad. Sci. USA *94*, 10919–10924.

Radhakrishnan, N., Yadav, S.P., Sachdeva, A., Pruthi, P.K., Sawhney, S., Piplani, T., Wada, T., and Yachie, A. (2011). Human heme oxygenase-1 deficiency presenting with hemolysis, nephritis, and asplenia. J. Pediatr. Hematol. Oncol. *33*, 74–78.

Reddy, V.P., Chinta, K.C., Saini, V., Glasgow, J.N., Hull, T.D., Traylor, A., Rey-Stolle, F., Soares, M.P., Madansein, R., Rahman, M.A., et al. (2018). Ferritin H deficiency in myeloid compartments dysregulates host energy metabolism and increases susceptibility to *Mycobacterium tuberculosis* infection. Front. Immunol. *9*, 860.

Regev, D., Surolia, R., Karki, S., Zolak, J., Montes-Worboys, A., Oliva, O., Guroji, P., Saini, V., Steyn, A.J., Agarwal, A., and Antony, V.B. (2012). Heme oxygenase-1 promotes granuloma development and protects against dissemination of mycobacteria. Lab. Invest. *92*, 1541–1552.

Ridley, D.S., and Ridley, M.J. (1987). Rationale for the histological spectrum of tuberculosis. A basis for classification. Pathology *19*, 186–192.

Rockwood, N., Costa, D.L., Amaral, E.P., Du Bruyn, E., Kubler, A., Gil-Santana, L., Fukutani, K.F., Scanga, C.A., Flynn, J.L., Jackson, S.H., et al. (2017). *Mycobacterium tuberculosis* induction of heme oxygenase-1 expression is dependent on oxidative stress and reflects treatment outcomes. Front. Immunol. *8*, 542.

Salgame, P. (2011). MMPs in tuberculosis: granuloma creators and tissue destroyers. J. Clin. Invest. *121*, 1686–1688.

Scharn, C.R., Collins, A.C., Nair, V.R., Stamm, C.E., Marciano, D.K., Graviss, E.A., and Shiloh, M.U. (2016). Heme Oxygenase-1 regulates inflammation and mycobacterial survival in human macrophages during *Mycobacterium tuberculosis* infection. J. Immunol. *196*, 4641–4649.

Schindelin, J., Arganda-Carreras, I., Frise, E., Kaynig, V., Longair, M., Pietzsch, T., Preibisch, S., Rueden, C., Saalfeld, S., Schmid, B., et al. (2012). Fiji: an open-source platform for biological-image analysis. Nat. Methods *9*, 676–682.

Sellers, K., Fox, M.P., Bousamra, M., 2nd, Slone, S.P., Higashi, R.M., Miller, D.M., Wang, Y., Yan, J., Yuneva, M.O., Deshpande, R., et al. (2015). Pyruvate carboxylase is critical for non-small-cell lung cancer proliferation. J. Clin. Invest. *125*, 687–698.

Shiloh, M.U., Manzanillo, P., and Cox, J.S. (2008). *Mycobacterium tuberculosis* senses host-derived carbon monoxide during macrophage infection. Cell Host Microbe *3*, 323–330.

Silva-Gomes, S., Appelberg, R., Larsen, R., Soares, M.P., and Gomes, M.S. (2013). Heme catabolism by heme oxygenase-1 confers host resistance to Mycobacterium infection. Infect. Immun. *81*, 2536–2545.

Silva Miranda, M., Breiman, A., Allain, S., Deknuydt, F., and Altare, F. (2012). The tuberculous granuloma: an unsuccessful host defence mechanism providing a safety shelter for the bacteria? Clin. Dev. Immunol. *2012*, 139127.

Tenhunen, R., Marver, H.S., and Schmid, R. (1968). The enzymatic conversion of heme to bilirubin by microsomal heme oxygenase. Proc. Natl. Acad. Sci. USA *61*, 748–755.

Tzima, S., Victoratos, P., Kranidioti, K., Alexiou, M., and Kollias, G. (2009). Myeloid heme oxygenase-1 regulates innate immunity and autoimmunity by modulating IFN-beta production. J. Exp. Med. *206*, 1167–1179.

Wagener, F.A., Eggert, A., Boerman, O.C., Oyen, W.J., Verhofstad, A., Abraham, N.G., Adema, G., van Kooyk, Y., de Witte, T., and Figdor, C.G. (2001). Heme is a potent inducer of inflammation in mice and is counteracted by heme oxygenase. Blood *98*, 1802–1811.

Yachie, A., Niida, Y., Wada, T., Igarashi, N., Kaneda, H., Toma, T., Ohta, K., Kasahara, Y., and Koizumi, S. (1999). Oxidative stress causes enhanced endothelial cell injury in human heme oxygenase-1 deficiency. J. Clin. Invest. *103*, 129–135.

## STAR★METHODS

### KEY RESOURCES TABLE

| REAGENT or RESOURCE | SOURCE | IDENTIFIER |
|---|---|---|
| Antibodies | | |
| HO-1 polyclonal (rabbit) | Enzo Life Sciences | Cat# ADI-SPA-895-F; RRID: AB_10618757 |
| Anti HO-1 mAb | Abcam | Cat# ab13248; RRID: AB_2118663 |
| NRF-2 polyclonal (rabbit) | Santa Cruz Biotech. | Cat# sc-722; RRID: AB_2108502 |
| NOS-2 polyclonal (rabbit) | Santa Cruz Biotech. | Cat# sc-651: RRID: AB_2298577 |
| Arg-1 polyclonal (rabbit) | Santa Cruz Biotech. | Cat# sc-20150; RRID: AB_2058955 |
| Histone H2A.X (goat) | Santa Cruz Biotech. | Cat# sc54607; RRID: AB_2118807 |
| MPO heavy chain (goat) | Santa Cruz Biotech. | Cat# sc34161; RRID: AB_2146338 |
| Neutrophil elastase (goat) | Santa Cruz Biotech. | Cat# sc9521; RRID: AB_2096537 |
| Goat anti-rabbit IgG H&L | Abcam | Cat# ab6721; RRID: AB_955447 |
| Alexa Fluor 555 Donkey anti-Goat IgG (H+L) | ThermoFisher Sci. | Cat# A21432; RRID: AB_2535853 |
| FITC HO-1, mAb (HO-1-2) | Enzo Life Sciences | Cat# ADI-OSA-111FI; RRID: AB_10621842 |
| BV711 anti-human CD14 mAb (M5E2), Conj-BV711 | BD Biosciences | Cat# 740773; RRID: AB_2740436 |
| V450 anti-human CD66b mAb (G10F5) | BD Biosciences | Cat# 561649; RRID: AB_10897169 |
| AF700 anti-human CD45 mAb (HI10) | BD Biosciences | Cat# 560566; RRID: AB_1645452 |
| PE-CF594 anti-human CD16 mAb (3G8) | BD Biosciences | Cat# 562293; RRID: AB_11151916 |
| PE-Cy5 anti-human CD206 mAb (19.2) | BD Biosciences | Cat# 551136; RRID: AB_394066 |
| Anti-human IgG4 | LifeSpan BioSciences | Cat# LS-C70325; RRID: AB_1655511 |
| Rabbit IgG, polyclonal - Isotype Control | Abcam | Cat# ab37415; RRID: AB_2631996 |
| APC anti-mouse IFN-$\gamma$ (XMG1.2) | Biolegend | Cat# 505810; RRID: AB_315404 |
| APC-eFluor® 780 anti-Mouse CD69 (H1.2F3) | eBioscience | Cat# 47-0691-82; RRID: AB_2573966 |
| eVolve 605 anti-Mouse CD4 (RM4-5) | eBioscience | Cat# 83-0042-42; RRID: AB_2574694 |
| Brilliant Violet 650 anti-mouse CD45.2 (104) | Biolegend | Cat# 109836; RRID: AB_2563065 |
| eVolve 605 anti-mouse CD11b (M1/70) | eBioscience | Cat# 83-0112-42; RRID: AB_2574700 |
| APC anti-mouse Ly-6G (1A8-Ly6g) | eBioscience | Cat# 17-9668-82; RRID: AB_2573307 |
| PE anti-mouse Ly-6G/Ly-6C (RB6-8C5) | Biolegend | Cat# 108408; RRID: AB_313373 |
| APC-eFluor 780 anti-mouse F4/80 (BM8) | eBioscience | Cat# 47-4801-82; RRID: AB_2735036 |
| PerCP anti-mouse Ly-6C (HK1.4) | Biolegend | Cat# 128028; RRID: AB_10897805 |
| eFluor 450 Fixable Viable Dye | eBioscience | Cat# 65-0863-14 |
| Bacterial and Virus Strains | | |
| *Mycobacterium tuberculosis* H37Rv | ATCC | N/A |
| Biological Samples | | |
| Blood and lung tissues from TB patients | King DinuZulu Hospital Complex | Study ID# BE 019/13 |
| Chemicals, Peptides, and Recombinant Proteins | | |
| ECL western blotting reagent | GE Healthcare | Cat# RPN2106 |
| RNeasy Plus Mini Kit | QIAGEN | Cat# 74134 |
| GeneChip Mouse Genome 430 2.0 Array | ThermoFisher Scientific | Cat# 900497 |
| Middlebrook 7H9 liquid medium | Fisher | Cat# DF0713-17-9 |
| Middlebrook 7H11 agar | Fisher | Cat# DF0838-17-9 |
| SSO Advanced SYBR Green Super Mix | Bio-Rad | Cat# 1725271 |
| iScript cDNA Synthesis Kit | Bio-Rad | Cat# 1708891 |
| Collagenase-D | Sigma | Cat# 11088866001 |
| Collagenase-B | Sigma | Cat# 11088815001 |

(*Continued on next page*)

*Continued*

| REAGENT or RESOURCE | SOURCE | IDENTIFIER |
|---|---|---|
| 4-Amino-5-Methylamino-2′,7′-Difluorofluorescein (DAF-FM) | ThermoFisher Sci. | Cat# D23841 |
| Dihydroethidium (DHE) | ThermoFisher Sci. | Cat# D1168 |
| Critical Commercial Assays | | |
| Mouse cytokine 23-plex | Bio-Rad | Cat# M60009RDPD |
| BCA Protein assay kit | ThermoFisher Sci. | Cat# 23227 |
| Deposited Data | | |
| Raw microarray data | This paper | ArrayExpress: E-MTAB-7221 |
| Experimental Models: Organisms/Strains | | |
| Human subjects (TB patients and healthy controls) | King DinuZulu Hospital Complex | Study ID# BE 019/13 |
| *Mycobacterium tuberculosis* H37Rv | ATCC | https://www.atcc.org/products/all/27294.aspx |
| global HO-1–deficient mice (HO-1−/−) on a C57BL/6 × FVB background and their HO-1+/+ (wild-type) littermates | Provided by co-author (A.A.) | Hull et al., 2015 |
| myeloid-specific HO-1−/− mice (referred to as HO-1LysM−/−) and HO-1 flox mice on C57BL/6 that lack the LysM-Cre construct (HO-1LysM+/+) | Provided by co-author (A.A.) | Hull et al., 2015 |
| Oligonucleotides | | |
| Mouse HO-1 For 5′-ggtgatggcttccttgtacc-3′ and 5′-agtgaggcccataccagaag-3′ | This paper | N/A |
| Software and Algorithms | | |
| FACS Diva Software | BD Biosciences | http://www.bdbiosciences.com/us/instruments/clinical/software/flow-cytometry-acquisition/bd-facsdiva-software/m/333333/overview |
| FlowJo 10 | FlowJo | https://www.flowjo.com/solutions/flowjo/downloads |
| NDPItools | N/A | https://www.imnc.in2p3.fr/pagesperso/deroulers/software/ndpitools/ |
| GraphPad Prism 6.0 | GraphPad Software | https://www.graphpad.com/scientific-software/prism/ |

## LEAD CONTACT FOR REAGENT AND RESOURCE SHARING

Further information and requests for resources and reagents should be directed to Lead Contact (adrie.steyn@ahri.org or asteyn@uab.edu).

## EXPERIMENTAL MODEL AND SUBJECT DETAILS

### Human Subjects

The study was approved by the University of KwaZulu-Natal Biomedical Research Ethics Committee (Class approval study number BCA 535/16). Patients undergoing lung resection for TB (Study ID: BE 019/13) were recruited from King DinuZulu Hospital Complex, a tertiary center for TB patients in Durban, South Africa. In Durban, South Africa, *Mtb*-infected human lung tissues are routinely obtained following surgery for removal of irreversibly damaged lobes or lungs (bronchiectasis and/or cavitary lung disease). Written informed consent was obtained from all participants. All patients undergoing lung resection for TB had completed a full 6-9-month course of anti-TB treatment, or up to 2 years of treatment for drug-resistant TB. Patients were assessed for extent of pulmonary disease (cavitation and or bronchiectasis) via high-resolution computed tomography. The fitness to withstand a thoracotomy and lung resection of each patient was determined by Karnofsky score, six-minute walk test, spirometry and arterial blood gas. Assessment of patients with massive hemoptysis included their general condition, effort tolerance prior to hemoptysis, arterial blood gas measurement, serum albumin level and an HRCT of the chest. Pneumonectomy specimens that were excised for pulmonary TB showed cavitational disease and tubercles and were selected for assessment. On gross assessment all pneumonectomies were bronchiectatic, hemorrhagic, variably fibrotic and atelectatic and contained tubercles that measured 2-5 mm in size. One specimen demonstrated a tuberculous cavity; Aspergillomas were not present. Please refer to the Table S1 for specific details on each patient.

## Animals

Global HO-1–deficient mice (HO-1−/−) on a C57BL/6 × FVB background and their HO-1+/+ (wild-type) littermates (Hull et al., 2015; Poss and Tonegawa, 1997) and myeloid-specific HO-1−/− mice (referred to as HO-1LysM−/−) and HO-1 flox mice on C57BL/6 that lack the LysM-Cre construct (HO-1LysM+/+) (Hull et al., 2015; Jais et al., 2014) were used in the study. Age-matched mice (8-12 weeks old) were housed under animal BSL-3 conditions following *Mtb* H37Rv infection and monitored daily. All the animals were maintained in pathogen free facility and animal maintenance and all procedures followed protocols approved by the Institutional Animal Care and Use Committee of the University of Alabama at Birmingham.

## Bacterial Strains

For animal infection studies, *Mycobacterium tuberculosis* H37Rv was used as descried (Reddy et al., 2018). *Mtb* was grown at 37°C with shaking in BD Difco Middlebrook (MB) 7H9 (broth) or standing on MB7H11 (agar) media supplemented with 0.2% glycerol, ADS (Albumin, Dextrose, NaCl) with 0.02% tyloxapol.

# METHOD DETAILS

## Infection of Mice

*Mtb* H37Rv was grown to mid-log phase in Middlebrook 7H9 liquid medium (Difco, Detroit, MI, USA) containing 0.2% glycerol, and 0.02% Tyloxapol supplemented with ADS (Albumin, Dextrose, Saline). Cells were washed and re-suspended in PBS containing 0.02% Tyloxapol to give a final inoculum of 5x104 CFU in 50 μl. Mice received intratracheal instillation of 50μl of inoculum or PBS. Animals were observed for survival (n = 10) or sacrificed at the indicated time points (n = 4 per time point). Bronchoalveolar lavage (BAL) fluid and lung leukocyte infiltrates were isolated in sterile PBS as described previously (16). For microarray, calculation of bacillary burden, cytokine analysis, histopathological and flow cytometric analysis of lung tissue were performed as described below.

## Microarray and Pathway Analysis

Blood monocytes were isolated from mice infected with *Mtb* H37Rv. Total RNA was harvested 24 hours post-infection for cDNA preparation using QIAGEN protocols and reagents. cDNA was prepared using protocols recommended by Affymetrix and reagents obtained from Invitrogen. Biotin labeled-cRNA preparation was done using protocols and reagents from Affymetrix. The labeled-cRNA hybridized to AffymetrixMouse Genome 430 2.0 GeneChip® arrays (Cat. # 900497). Pathway analysis was done using Ingenuity Pathways Analysis (IPA). The results were ordered by -log10 of the p value of the hypergeometric distribution.

## Determination of Bacterial Burden

To determine the number of viable *Mtb* in the lung and spleen of infected mice, organs were removed aseptically at specified time points. Part of the tissue was homogenized in 0.02% Tween-80/PBS. Viable *Mtb* were determined as CFU by performing serial dilutions of the tissue homogenates in PBS and plating onto Middlebrook 7H11 agar plates (Difco Laboratories, Detroit, MI, USA) supplemented with ADS (Albumin, dextrose, and saline) and 50 μg each of Carbenicillin and Cycloheximide antibiotics (Sigma-Aldrich, St. Louis, MO, USA). The plates were incubated at 37°C and colonies were counted after 3-4 weeks. The plates were further incubated for an additional 2 weeks to ensure detection of slower growing *Mtb*. The number of CFU is expressed as cfu per lung/spleen from four individual mice for each time point.

## Isolation of BAL fluid and Cytokine ELISA

After euthanizing animal, trachea was exposed and a Safelet Cath (22 g × 1″, Exel) was inserted into the trachea and 1 mL of PBS was instilled and aspirated. The lavage samples were centrifuged at 2,500 × g for 5 min at 4°C to pellet the cells and the supernatant was stored until further analysis. Cytokine levels were determined using Bio-Plex ProTM Mouse Cytokine 23-Plex, Group-1 (BIO-RAD, Hercules, CA). Individual cytokine concentrations were measured and expressed in pg/ml as per manufacturer instructions.

## Western Blotting

*Mtb*-infected mouse lungs were lysed in RIPA buffer containing protease inhibitors (cOmplete Tablets, Roche) using a dounce homogenizer. Protein was quantified using the BCA protein assay kit (Thermo Scientific). 20 μg of total protein was resolved on a 4%–15% gradient SDS-PAGE gel (Bio-Rad) and transferred to a PVDF membrane. Membranes were blocked in 5% nonfat dry milk in PBS-T for 1 hr and then incubated with a primary antibody for HO-1 (ADI-SPA-895-F, Enzo Life Sciences, Farmingdale, NY) and Nrf-2 (SC-722, Santa Cruz Biotechnology, Dallas, TX.) followed by a peroxidase conjugated secondary antibody (ab6721, Abcam, Cambridge, MA). HRP activity was detected using ECL Western Blotting reagent (Amersham).

## RNA extraction and real-time PCR from lung tissues

Total RNA from lungs of *Mtb*-infected HO-1+/+ and HO-1−/− mice was extracted using RNeasy Plus mini kit as per manufacturers recommendations (QIAGEN). Genomic DNA was removed by treatment of RNA samples with RNase-free DNase I (Thermo Scientific) for 15 min at RT followed by 30 min at 37°C. One microgram of total RNA was used to generate cDNA by using the iScript cDNA

synthesis Kit (Bio-Rad). Quantitative real-time PCR was performed using SSO Advanced SYBR Green Supermix (Bio-Rad). The relative gene expression was normalized to mouse GAPDH as an internal control.

### Mouse Histopathology

Histological sections were stained with H&E or trichrome stain for evaluation of pathology and fibrosis, respectively. Briefly, samples of lung and spleen were aseptically removed and fixed in 10% buffered formalin and embedded in paraffin. Five-micrometer sections were stained with hematoxylin-eosin or the Masson trichrome method using standard procedures

### Flow Cytometry of Mice Immune Cell Isolates

Immune cells were isolated from lung tissue as described (16), in a BSL-3 facility using BSL-3 practices and procedures. Briefly, to reduce contamination of isolated lung cells with blood, the pulmonary circulation was perfused with PBS via the right ventricle following euthanasia and thoracotomy. Airway lavage was performed three times with 0.8 mL of PBS each. Infiltrating leukocytes were isolated from minced lung tissue by treatment with collagenase-B (2 mg/ml, Roche) and DNase I (0.02 mg/ml, Sigma) in Iscove's modified Dulbecco's medium (IMDM) supplemented with 1 mM sodium pyruvate, 2 mM L-glutamine, 10 μg/ml penicillin-streptomycin, 25 μM 2-mercaptoethanol and 0.1 mM non-essential amino acids (Life Technologies) at 37OC for 30 min. This was followed by the addition of an equal volume of IMDM containing 20% FBS. Cell suspensions were filtered using 40-μm cell strainer, washed with PBS, counted and fixed with buffered formalin. After fixation, the cells were washed in PBS and pretreated at 4°C for 20 minutes in FACS staining buffer (PBS + 3% FBS) containing 2.0 μg/ml of the mAb 2.4G2 to block Fc-mediated binding of subsequent antibodies (BD PharMingen, Franklin Lakes, NJ). These cells were then stained to identify and characterize immune cell populations using fluorescence conjugated antibodies directed against the cell surface markers for 30 min at 4°C (all antibodies were purchased from eBioscience, San Diego, CA). For detecting the frequency of IFN-γ-secreting cells, cells were cultured in RPMI-1640 containing 5 ng/ml PMA and 500 ng/ml Ionomycin along with 10 μg/ml GolgiPlug protein transport inhibitor (BD Biosciences, Sparks, MD), at 37°C for 3 hours. Cells were then harvested, fixed with buffered formalin and permeabilized using a BD cytofix/cytoperm fixation/permeabilization kit (BD Biosciences) and then stained with FITC-labeled 53-6.7 antibody directed against IFN-γ. Cells were washed twice with PBS before analysis. Flow cytometry acquisitions and analyses were carried out using Becton Dickinson LSR II with FACS Diva software (BD Biosciences, San Jose, CA). Data were further analyzed using FlowJo 10 (Tree Star, Ashland, OR).

### Human Lung Cell Isolation and Flow Cytometry

Lung tissue was washed with HBSS to remove excess blood and cut into small sections approximately 0.5 mm thick with a sterile scalpel. The sections were washed 3 times with RPMI 1640 medium containing 10% fetal calf serum. To isolate interstitial cells, lung sections were digested with 0.5 mg/ml type collagenase D (Sigma) and 40 units/ml DNAase I (Roche). Tissue was then mechanically dissociated using the GentleMacs system (lung program, Miltenyi) at 37°C for 60 minutes followed by a second round of dissociation. Cells were then washed twice in medium, strained through a 70 μM cell strainer. Isolated lung cells were surface stained with mAbs directed against CD14 (M5E2; BD Biosci.), CD66b (G10F5, BD Biosci.), CD45 (HI10; BD Biosci.), CD16 (3G8; BD Biosci.), CD206 (19.2; BD Biosci.) at 4°C for 30 minutes followed by two washes with PBS. To detect intracellular HO-1 and iNOS levels, the surface labeled cells were fixed and permeabilized (Cytofix/CytoPerm, BD) and stained with FITC labeled conjugated HO-1 (HO-1; Enzo) or anti-iNOS (Novous Biologicals) at 4°C for 30 min followed by two washes with PBS. For ROI/RNI detection, following cell surface labeling, cells were washed and stained either with 25 μM DHE or 25 μM DAF-FM (Life technologies) at 37°C for 30 minutes followed by two PBS washes. Data acquisition was performed on a BD Biosciences Fortessa flow cytometer and analysis was performed with Flowjo Vx.0.7.

### Human Lung Tissue Immunohistochemistry

Human lung tissues were cut into 2 μm thick sections were mounted on charged slides and heated at 56°C for 15 min. Mounted sections were dewaxed in xylene followed by rinse in 100% ethanol and 1 change of SVR (95%). Slides were then washed under running water for 2 min followed by antigen retrieval via Heat Induced Epitope Retrieval (HIER) in Tris-sodium chloride (pH 6.0) for 30 min. Slides were then cooled for 15 min and rinsed under running water for 2 min. Endogenous peroxide activity was blocked using 3% hydrogen peroxide for 10 min at room temperature (RT). Slides were then washed in PBST and blocked with protein block (Novolink) for 5 min at RT. Sections were incubated with primary antibodies for HO-1 (ab13248, Abcam; 1:100 dilution), Nrf-2 (sc-722, Santa Cruz Biotechnology, 1:100), Arg-1 (sc-20150, Santa Cruz Biotechnology, Inc., 1:100 dilution), NOS2 (sc-651, Santa Cruz Biotechnology, 1:100 dilution), MPO heavy chain (sc-34161, SantaCruz Biotechnology, 1:100), Histone H2A.X (sc-54607, SantaCruZ Biotechnology, 1:100) and Neutrophil elastase (sc9521, SantaCruz Biotechnology) followed by washing and incubation with either HRP anti-rabbit IgG HRP (ab6721, abcam), anti-Goat IgG (H+L)-Alexa Fluor 555 or the polymer (Novolink) for 30 min at RT. Slides were then washed and stained with DAB for 5 min, washed under running water and counterstained with hematoxylin for 2 min. Slides were rinsed under running water, blued in 3% ammoniated water for 30 s, washed under water, dehydrated and mounted in Distyrene Plasticiser Xylene (DPX). For isotype control sections, a similar protocol was followed and either IgG4 (LS-C70325/27332) or rabbit IgG (ab37415, Abcam) was used (at the same concentration/dilution as the primary antibodies) in place of the primary antibodies (isotype control). For neutrophil staining, after secondary staining, slides were washed, dehydrated and mounted using CytoSeal-60 (ThermoFisher Sci.) and images were taken on Nikon A1R confocal microscope.

### Spatial image analysis of HO-1 distribution within human TB granulomas

Pathology images were captured at 10x magnification on a Hamamatsu NanoZoomer 2.0RS slide scanner. The resulting image files were converted to JPEG format (lossless compression) with NDPItools (https://www.imnc.in2p3.fr/pagesperso/deroulers/software/ndpitools/) for further analysis. Background over-staining was subsequently removed in in FIJI (FIJI Is Just ImageJ) using the Subtract Background plugin (settings: rolling ball radius of 50 pixels, light background, separate colors). Following that, deconvolution was performed in using the Color Deconvolution plugin FIJI (using custom trained vectors for H-DAB staining) (Schindelin et al., 2012). Anaconda/Python scripts calling the numpy, scipy, skimage, pandas, seaborn and matplotlib libraries were used for further analysis. A sliding window of 10001 and 1001 pixels (necrotic lesions and NNGI granulomas respectively) was used to calculate running averages for DAB stain intensity projected on the major axis (normalized to 1.0) of a rectangular region traversing each granuloma, lesion or appropriate control region. The same size rectangular region was used for each necrotic lesion and granuloma, respectively.

## QUANTIFICATION AND STATISTICAL ANALYSIS

Statistical analyses were performed using unpaired, two tailed Student's t test and the data were expressed as mean ± SEM, unless indicated, using GraphPad Prism 6 (GraphPad Software, Inc., La Jolla, USA). Statistical significance was defined as: $^*p < 0.05$, $^{**}p < 0.01$, $^{***}p < 0.001$. Statistical test, method of error calculation and significance for each figure can be found in the respective figure legend.

## DATA AND SOFTWARE AVAILABILITY

The accession number for the raw and processed microarray data reported in this paper is ArrayExpress: E-MTAB-7221 (www.ebi.ac.uk/arrayexpress).

High resolution immunostaining figures will be provided upon request or can be downloaded at: https://www.ahri.org/scientist/adrie-steyn/ (Accessed: January 1, 2019). Please contact Dr. Adrie J.C. Steyn (adrie.steyn@ahri.org or asteyn@uab.edu).

Cell Reports, Volume *25*

## Supplemental Information

# Microanatomic Distribution of Myeloid Heme Oxygenase-1 Protects against Free Radical-Mediated Immunopathology in Human Tuberculosis

**Krishna C. Chinta, Md. Aejazur Rahman, Vikram Saini, Joel N. Glasgow, Vineel P. Reddy, Jeremie M. Lever, Shepherd Nhamoyebonde, Alasdair Leslie, Ryan M. Wells, Amie Traylor, Rajhmun Madansein, Gene P. Siegal, Veena B. Antony, Jessy Deshane, Gordon Wells, Kievershen Nargan, James F. George, Pratistadevi K. Ramdial, Anupam Agarwal, and Adrie J.C. Steyn**

## Supplemental Figures.

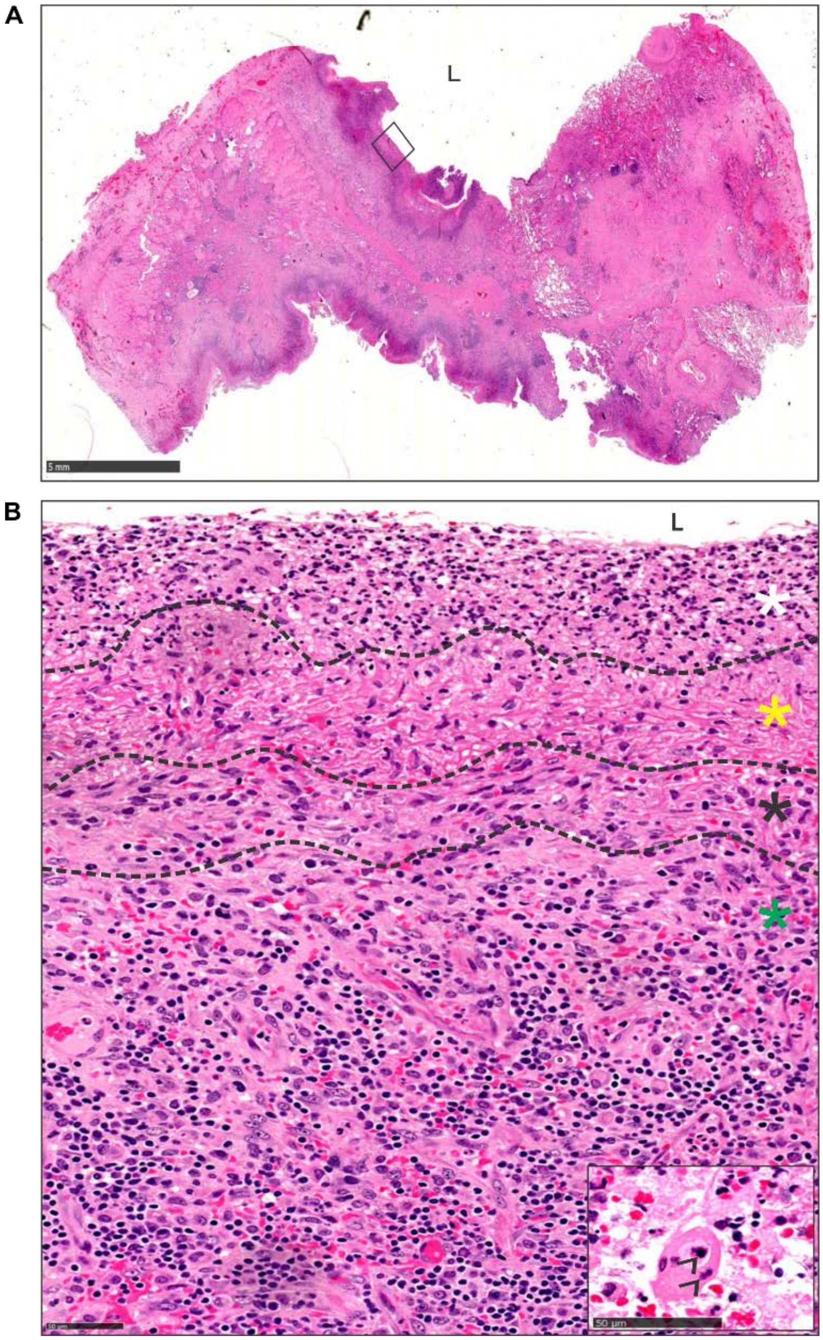


**Figure S1. Histomorphology of necrotising granulomatous inflammation: cavity wall. Related to Figure 1.** **(A)** Low power H&E demonstration of a tuberculous cavity wall with selected focus (rectangle; Fig. 1) and (**B**) High power spatial demonstration thereof. (B) Adluminal inflammatory cells composed of neutrophils and cellular debris (white asterisk) and focal giant cells (arrow) was juxtaposed to hypocellular fibrinoid necrosis (yellow asterisk). This abutted confluent granulomatous inflammation (black asterisk) composed predominantly of epithelioid histiocytes and scattered lymphocytes. This was bordered by granulation tissue (green asterisk). (L = lumen). Inset: High power demonstration of phagocytic giant cell with intracytoplasmic phagocytized neutrophils (arrowheads).

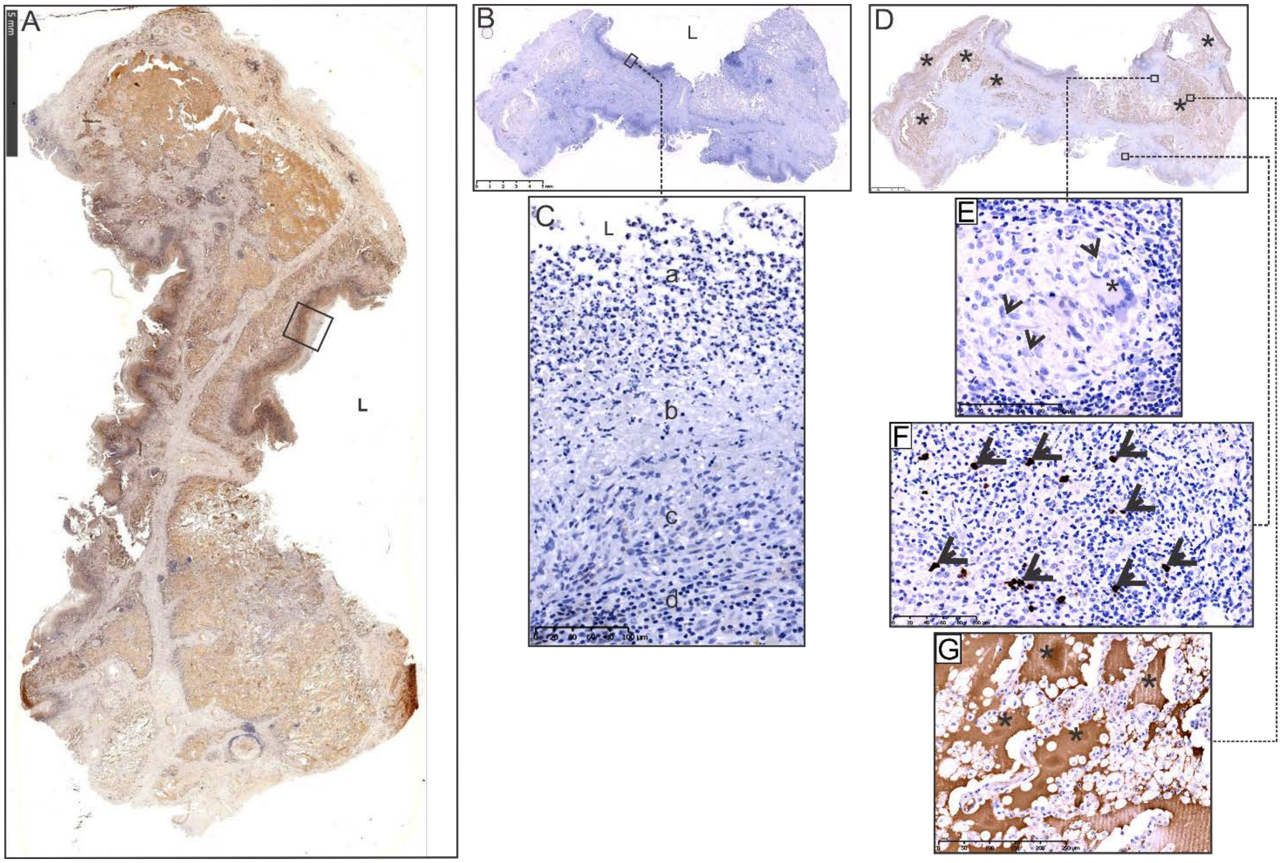


**Figure S2. HO-1 staining of cavity wall and isotype control staining in human TB lung tissue. Related to Figure 1 and 2.** Low power depiction of (**A**) HO-1 staining of tuberculous cavity wall with selected focus (rectangle) (L = lumen) and **(B)** Antibody control. (**C**) High power depiction of the selected focus highlighting the absence of staining in the wall of the cavity (L = lumen, a, b, c and d represent adluminal suppurative/karyorrhectic, fibrinoid necrosis, granulomatous and granulation tissue zones, respectively). (**D**) IgG4 was employed as the isotype control. Low power whole mount depiction of lgG4 staining in the tissue section. (**E**) High power demonstration of granuloma confirming immunonegative epithelioid histiocytes (black arrows) and a Langhans giant cell (asterisk). Note the lgG4 immunopositive plasma cells (**F**, arrows) in the granulation tissue layer serving as a positive, in-built isotype control response, and surrounding stromal immunonegative endothelial, mesenchymal and lymphoid cells. Additionally, as expected, intra-alveolar blood/serum products demonstrate immunopositivity (**G**, * in C, normal serum lgG4 level is 8-140 mg/dL). In contrast, the viable intra-alveolar and interstitial cellular components were immunonegative. The latter cells have clear cytoplasm and hematoxyphylic (blue) nuclei because of hematoxylin counterstaining.

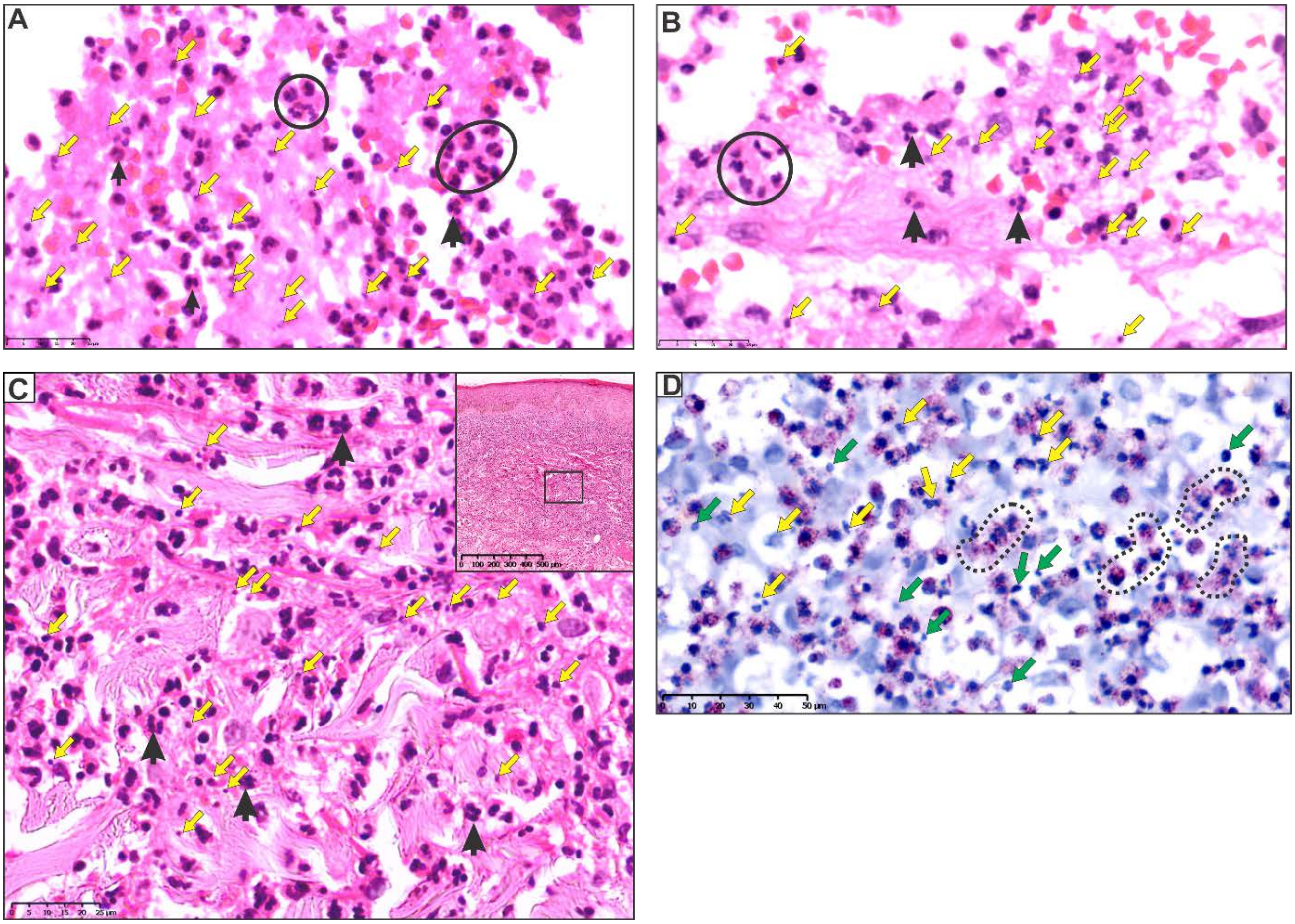


**Figure S3**. **Karyorrhexis; Histomorphological and histochemical demonstration of neutrophilic degradation. Related to Figure 1 and 2**. Sections of the (**A**) adluminal suppurative/karyorrhectic zone (in FigureS2A) (**B**) abscess contents and (**C**) Sweet syndrome to demonstrate the histomorphological features of karyorrhexis, characterized by nuclear fragmentation. Admixed aggregated (circle) and single (black arrows) intact neutrophils and nuclear (karyorrhectic) nuclear debris in the adluminal layer of the cavity wall. Similar histomorphological features are seen in two other diseases representing positive controls (B, C) in which karyorrhexis is a characteristic histomorphological attribute. Contents of abscess (B) highlighting admixed aggregated (circle) and single (black arrows) intact neutrophils and nuclear debris (karyorrhexis). Low power view of Sweet syndrome (neutrophilic dermatosis) demonstrating a dense cellular dermal infiltrate and high-power demonstration (C) of selected focus (rectangle, C) of intact neutrophils (black arrows) and nuclear fragments (yellow arrows). (**D**) Histochemical demonstration of neutrophil degeneration using chloroacetate esterase [Leder]) stain. Low power demonstration of viable neutrophils containing brightly positive rose-pink granules (dotted line oval shapes), degenerate trinucleate neutrophils (yellow arrows), and nuclear debris (green arrows) lacking chloroacetate esterase positive granules. Degenerate neutrophils lose their enzymatic cellular function, which is highlighted by degranulation and the loss of chloroacetate esterase-stained cytoplasmic granules in tissue sections.

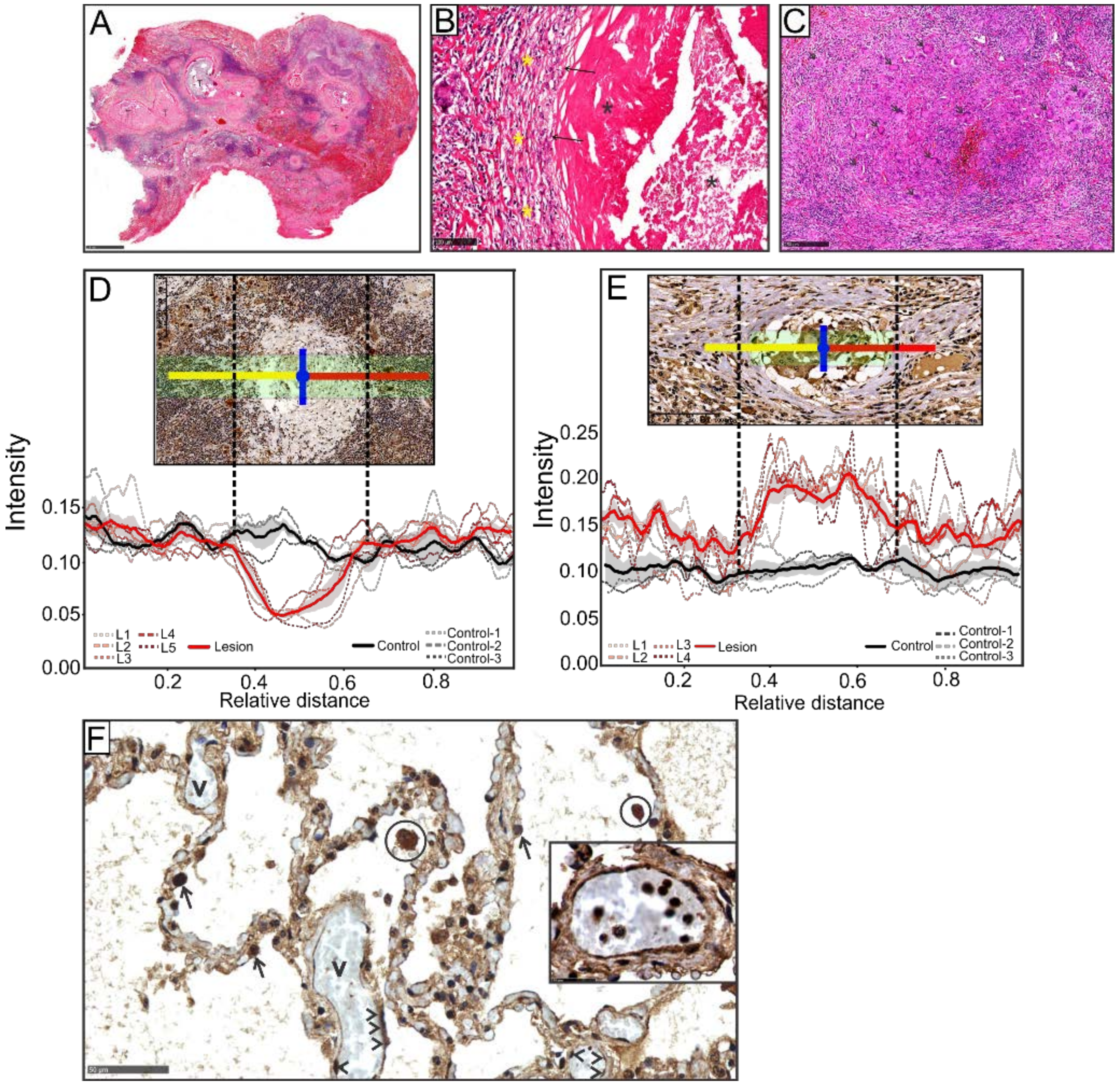


**Figure S4, Histomorphology and HO-1 distribution in granulomas and healthy tissues. Related to Figure 2.** (**A**) H&E staning of sections of lung parenchyma demonstrating congestion, hemorrhage and tubercles (T) on low power characterized by (**B**) necrotizing granulomatous inflammation with central caseative necrosis (black asterisks) surrounded by a fibro-inflammatory response consisting of granulomas (arrows) and vascularized lamellar fibrosis (yellow asterisks) on high power. (**C**) Medium power H&E depiction of confluent organized granulomas composed of Langhans giant cells (arrows), epithelioid histiocytes and lymphocytes. Decreased HO-1 levels within caseous necrotic lesions (**D**) and increased HO-1 levels in non-necrotic granulomatous inflammatory (NNGI) lesions (**E**). Representative images corresponding to caseous necrotic and NNGI are included for purposes of clarity; the scale does not correspond to the relative distance scale in the graphs. DAB stain intensity relative to a masked region of fixed size is plotted for five individual lesions (L) (dotted/dashed red series) and three control regions (dotted/dashed grey series) (**D**). Four lesions and three control regions were examined in **E**. Combined plots for granulomatous (red) and control (black) are indicated with confidence bands (shaded regions) at a confidence interval of 0.68. (**F**) HO-1 positive alveolar pneumocytes (arrows), endothelial cells in interstitial vessels (arrowheads) and focal intra-alveolar histiocytes (circles). Inset: HO-1 positive circulating intravascular neutrophils. V = vessel (blood).

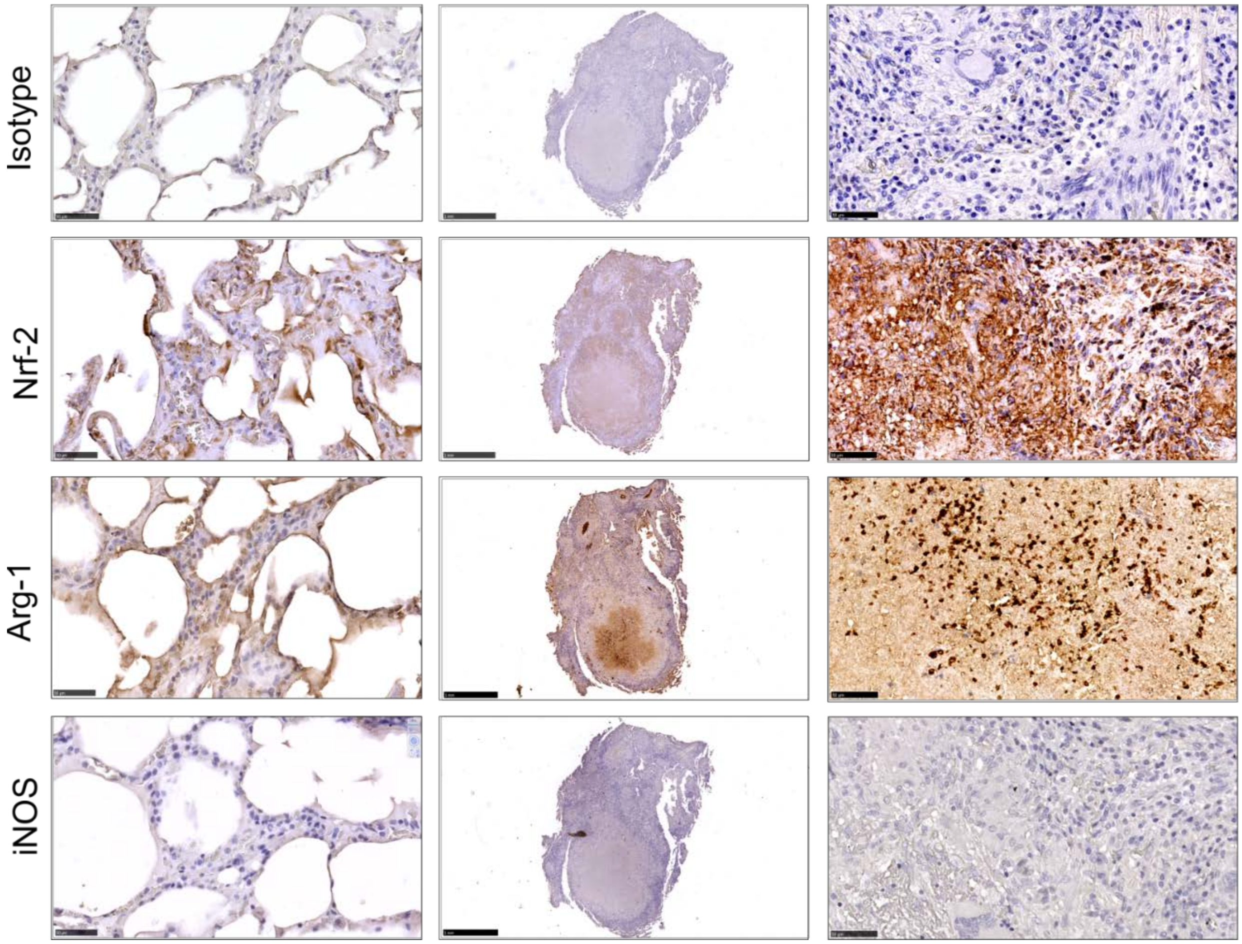


**Figure S5. Nrf-2, iNOS and Arg-1 are produced in human TB lung lesions, Related to Figure 2.** Representative tissue sections showing immunohistological detection of Nrf-2, Arg-1 and iNOS levels in diseased and healthy lung regions of TB patients (Images are represented at either 2x or 40x magnification). An immunonegative rabbit IgG isotype control is shown (isotype).

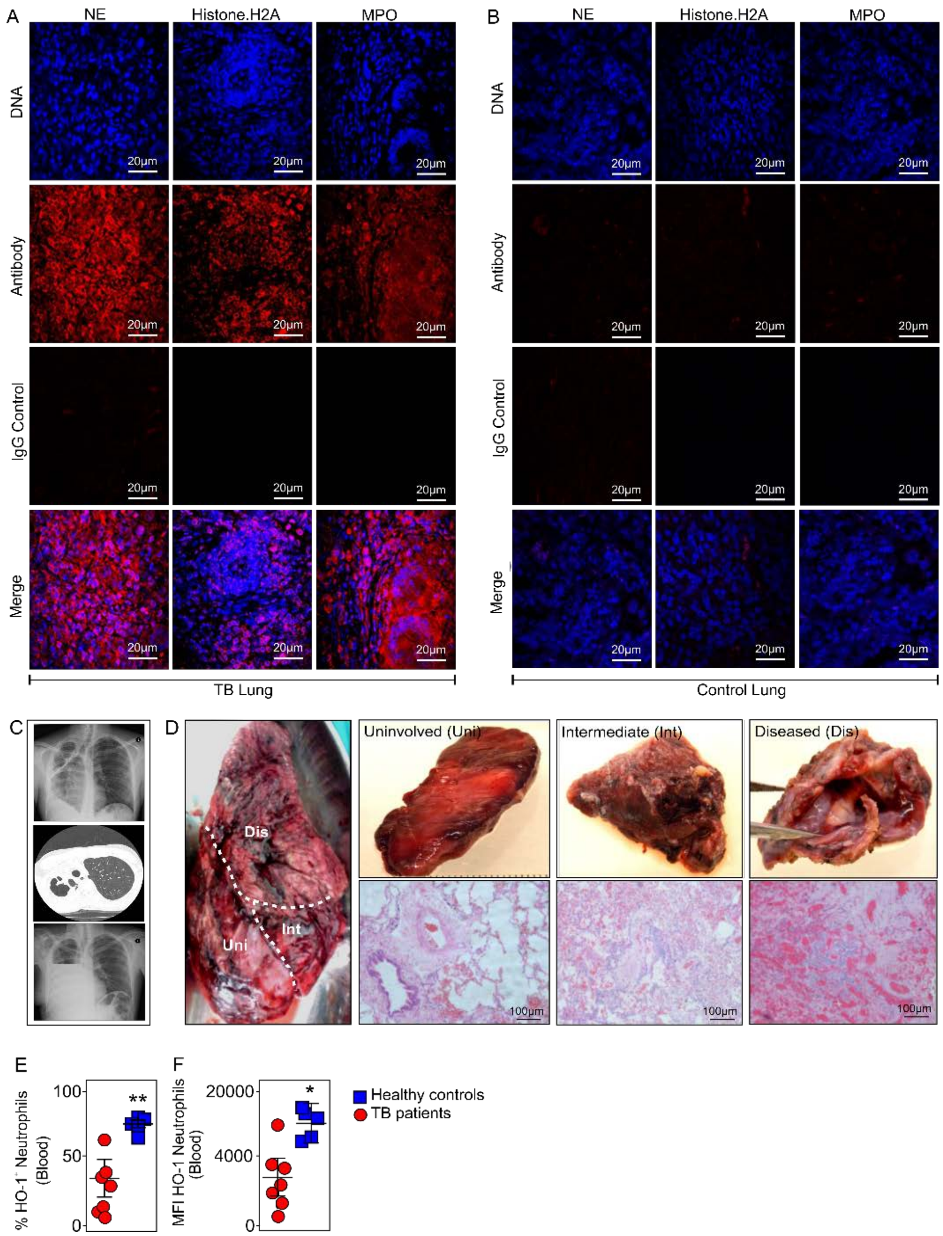


**Figure S6. Distinct pathological regions, neutrophil accumulation in human TB lungs and HO-1 levels in the blood of TB patients. Related to Figure 3.** Representative lung tissue sections showing immune-fluorescence detection of MPO, neutrophil elastase and histone.H2A which are markers of NETs in (**A**) TB patients and (**B**) control non-TB lung. (Images taken at 40x magnification). (**C**) Representative chest radiograph showing a shrunken right lung with a large cavity in the right upper lobe (top). The same patient underwent high-resolution computed tomography (HRCT) scan, which confirmed the cavity in the right upper lobe (middle). Second chest radiograph of same patient, post-pneumonectomy (bottom). (**D**) Resected lung of same patient and identification of distinct pathological regions by gross pathology and H&E staining (Images taken at 10x magnification). (**E**, **F**) HO-1 levels in the neutrophils ($CD11b^+CD66b^+CD16^+CD14^-CD3^-$) isolated from the blood of TB patients compared to healthy controls. (**E**) Percent HO-$1^+$ neutrophils in the blood of Tb patients compared to healthy controls (**F**) MFI of HO-1 in neutrophils.

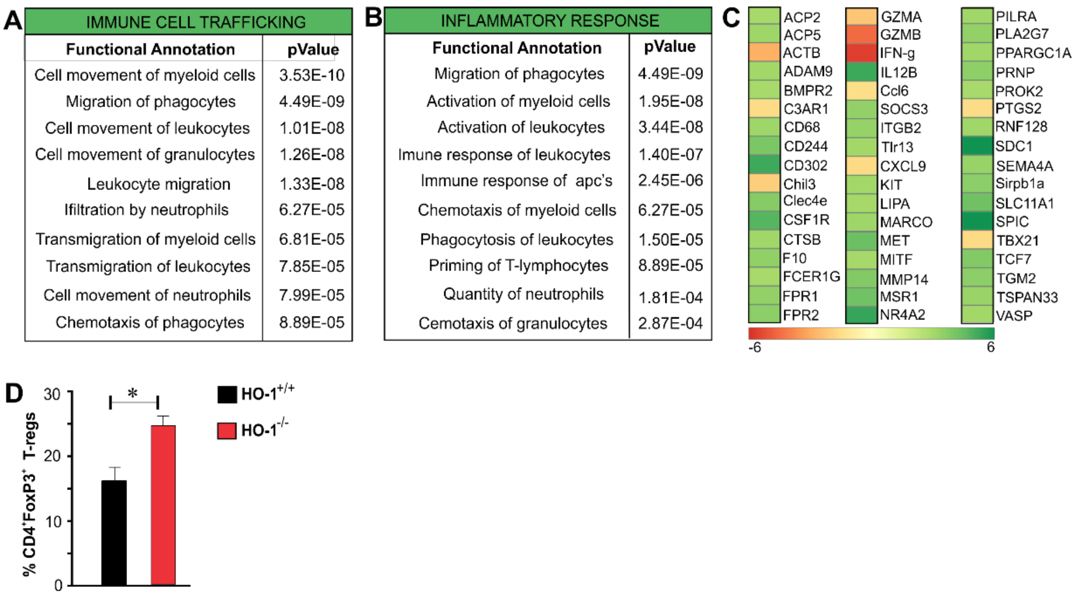


**Figure S7. Global transcriptomic profiles of blood monocytes from HO-1$^{+/+}$ and HO-1$^{-/-}$ mice following *Mtb* infection and increased T-reg accumulation in HO-1$^{-/-}$ mice**. **Related to Figure 5**. Ingenuity Pathways Analysis (IPA) pathway analysis showing the most significantly ranked pathways in the categories of (**A**) immune cell trafficking and (**B**) inflammatory responses. The results are ordered by -log10 of the *p*-value of the hypergeometric distribution. (**C**) Significantly regulated genes in the pathways mentioned in the A and B. Fold change greater than 1.5 was considered as significant. (**D**) Percent differences in CD4$^{+}$FoxP3$^{+}$ T-regs in the lungs of *Mtb*-infected HO-1$^{+/+}$ and HO-1$^{-/-}$ mice at 18 weeks post infection. n = 4 for each experiemnt. Statistical testing was performed using the unpaired Student's t-test. Data are represented as mean ± SEM. *p < 0.05.

**Supplemental Table.**

| ID | Age | Sex | TB Type | Type of Resection | Drug treated |
|---|---|---|---|---|---|
| 32 | 27 | Female | TB | LLP | Yes |
| 104 | 33 | Female | TB | RUL | Yes |
| 110 | 34 | Female | MDR TB | RLP | Yes |
| 30 | 39 | Female | TB | RUL | Yes |
| 23 | 40 | Female | XDR TB | RLP | Yes |
| 26 | 41 | Female | MDR-TB | LLP | Yes |
| 25 | 42 | Female | MDR-TB | LLP | Yes |
| 114 | 44 | Female | TB | LLP | Yes |
| 42 | 46 | Female | TB | RLP | Yes |
| 24 | 21 | Male | MDR-TB | RLP | Yes |
| 40 | 26 | Male | MDR TB | LUL | Yes |
| 37 | 27 | Male | TB | LUL | Yes |
| 27 | 31 | Male | TB | RLP | Yes |
| 107 | 33 | Male | TB | LUL | Yes |
| 115 | 35 | Male | TB | LUL | Yes |
| 103 | 39 | Male | TB | LLP | Yes |
| 102 | 40 | Male | TB | LLP | Yes |
| 38 | 41 | Male | MDR TB | RLP | Yes |
| 113 | 52 | Male | TB | LUL | Yes |
| 108 | 56 | Male | TB | LWL | Yes |
| 31 | 66 | Male | TB | RUL | Yes |

**Table S1. Clinical characteristics of human subjects. Related to Figure 1, 2,3 and Star Methods.** Males and female patients were recruited at King DinuZulu Hospital Complex, Durban, South Africa. Written consent was taken from all the patients. Before recruitment all the patients were subjected to tests described in Supplemental Experimental Procedures. Acronyms used: TB. Tuberculosis; LLP. Left Lung Pneumonectomy; RLP. Right Lung Pneumonectomy; RUL. Right Upper Lobectomy; LUL. Left Upper Lobectomy; LWL. Left Whole Lobectomy; MDR-TB. Multi Drug-Resistant TB; XDR-TB. Extensively Drug-Resistant TB.
